# Trends in the incidence and survival of cancer in individuals aged 55 years and older in the United States, 1975–2019

**DOI:** 10.1186/s12889-023-17571-x

**Published:** 2024-01-03

**Authors:** Junpeng Cui, Rongmei Ding, Haifeng Liu, Mingxiu Ma, Ruixue Zuo, Xun Liu

**Affiliations:** 1 Department of General Surgery, Shengjing Hospital of China Medical University, No. 36 Sanhao Street, Heping District, Shenyang, 110004 Liaoning China; 2 Department of Oncology, Shengjing Hospital of China Medical University, No. 36 Sanhao Street, Heping District, Shenyang, 110004 Liaoning China

**Keywords:** Cancer, Incidence rate, Older population, Cancer prevention, Survival rate

## Abstract

**Background:**

In ageing societies such as the United States, evaluating the incidence and survival rates of cancer in older adults is essential. This study aimed to analyse the incidence and survival rates of cancer in individuals aged 55 years or older in the United States.

**Methods:**

This retrospective study (1975–2019) was conducted using combined registry data from the Surveillance, Epidemiology, and End Results database. Data from the 9, 12, and 17 Registries (Nov 2021 Sub) datasets were used.

**Results:**

In 2019, the incidence of cancer in individuals older than 55 years and the overall population was 1322.8 and 382.1 per 100,000 population, respectively. From 2000 to 2019, the incidence of cancer in individuals older than 55 years showed a decreasing trend, whereas their five-year survival rates showed an increasing trend. The incidence of cancer in the 75–79 and 80–84 year age groups was the highest among all age groups.

**Conclusions:**

The incidence of colon cancer declined significantly, whereas that of intrahepatic bile duct cancer increased considerably. These trends may be due to increased screening for cancers with high incidence rates and improved control of the risk factors for cancer. Rapid development of targeted therapy and immunotherapy combined with early tumour detection may be an important reason for the improved survival rates.

**Supplementary Information:**

The online version contains supplementary material available at 10.1186/s12889-023-17571-x.

## Background

Cancer has a significant negative impact on life expectancy and health worldwide [1]. The incidence and mortality rates of cancer are rapidly increasing worldwide due to the ageing population and global population growth [2]. In 2019, the World Health Organization reported cancer as the leading cause of death in individuals younger than 70 years in the United States [2]. The reasons for the high incidence of cancer in the older population are complex [3]. In older individuals, the tissue microenvironment deteriorates owing to insufficient maintenance capacity during tissue regeneration, leading to inadequate control of abnormal or mutated cells, resulting in the development of cancer [4]. Several factors contribute to this process, including long-term exposure to external stimuli, accumulation of senescent cells, gene mutations, stem cell exhaustion, immune system decline, mitochondrial dysfunction, and chronic inflammation [5, 6]. In summary, the increased incidence of cancer in the older population can be attributed to age-related deterioration of physical functions, changes in the tissue microenvironment [7], and the influence of long-term pathogenic factors. The prevention and treatment of age-related cancers have increasingly shifted toward individualised and targeted approaches, including the treatment of specific pathogenic genes [6, 8, 9]. Thus, in the current scenario of an ageing global population, focusing on the incidence and survival of cancers in older adults allows for a better understanding of trends in cancer incidence and survival rates as well as a more intuitive assessment of the effects of cancer prevention and treatment.

The incidence of cancer considerably increases after the age of 55 years [3, 10]. Therefore, we used data from the Surveillance, Epidemiology, and End Results (SEER) database to investigate cancer incidence and survival rates among individuals aged 55 years or older to assess and analyse the current trends in the incidence and survival of cancers as well as the most common types of cancers among older adults in the United States.

## Methods

### Data source

This retrospective study was conducted using data from the SEER database [11], which collects data from multiple centres on cancer incidence and patient information, including race, sex, primary tumour site, tumour morphology, stage at diagnosis, first course of treatment, and survival status, from population-based cancer registries covering approximately 47.9% of the United States population.

### Selection and analysis of variables

The patient cohort was obtained from the SEER database, and patients aged 55 years or older were selected. Cancer cases were identified using the International Classification of Diseases for Oncology 3rd edition/WHO 2008 (ICD-O-3/WHO 2008) [12] recodes for all sites and malignant behaviours. Patients with only autopsy or death certificate data were excluded, as well as patients with unknown age. For the analysis and comparison of cancer incidence in the overall population and patients aged ≥ 55 years from 1975 to 2019, we used three data registries categorised into specific periods: 9 Registries, Nov 2021 Sub (1975–1991); 12 Registries, Nov 2021 Sub (1992–1999); 17 Registries, Nov 2021 Sub (2000–2019). We analysed the overall incidence of cancer from 1975 to 2019, compared the incidence rates across different age groups, and examined the incidence trend in individuals aged ≥ 55 years. Then, we utilised the 17 Registries, Nov 2021 Sub (2000–2019) dataset to analyse the incidence and survival rates for different cancers over the past 20 years in individuals aged ≥ 55 years, categorised according to age, sex and stage. ‘In situ’, ‘Localized’, ‘Regional’, and ‘Distant’ were used in summary stage 2000 (1998–2017) [13] to analyse the incidence of tumour stage from 2000 to 2017.

A total of 80 cancer sites were included in the study, spanning from 1975 to 2019. These cancer sites were classified using the International Classification of Diseases for Oncology 3rd edition/WHO 2008 and are listed in Supplement Table 5. These specific cancers were selected for analysis because they were included and recorded in the SEER database. Moreover, these cancers cover different parts of the body and represent a wide range of malignant tumours in clinical settings. By examining them collectively, we can gain insights into the overall occurrence and survival rates of cancer in the population.

### Statistical analysis

Age-adjusted incidence rates were calculated using the weighted proportions of the corresponding age groups in the 2000 United States standard population [14]. Percentage changes were calculated using the previous year as the reference point [15]. The annual percentage change (APC) in incidence rate over the past 20 years (2000–2019) was calculated using the weighted least squares method and data from the 17 Registries Nov 2021 Sub dataset. Survival time was defined as the duration in months from the date of diagnosis to the date of death. Period survival statistics were estimated for 1–5 years by combining recent conditional survival estimates for multiple cohorts. Survival rate, which excluded the risk of dying from other causes, was calculated [16]. Data were analysed using SEER*Stat software version 8.4.0.1, which was released on 15 April 2022 [17]. Plots were created using Office Excel 2021 Long Term Servicing Channel (Microsoft Corporation, USA). A P-value of < 0.05 was considered statistically significant.

### Ethics approval/consent

This article does not describe any study involving human participants or animals performed by any of the authors. Formal patient consent was not required in this study.

## Results

In this study, three SEER registries were integrated for analysis, including 6,847,425 patients aged 55 years or older. The patient population consisted of 3,143,989 women (45.91%) and 3,703,436 men (54.09%), with 80 cancer sites (Supplementary Table 1 and Supplementary Table 5) and a median age of 72 years. With 17 Registries Nov 2021 Sub dataset, in the last 20 years, prostate cancer, breast cancer, lung and bronchial cancer, colon and rectal cancers, and urinary bladder cancer were the most prevalent cancers with high age-adjusted incidence rates.

### Annual incidence of cancer in individuals aged ≥ 55 years from 1975 to 2019

**Fig. 1:**
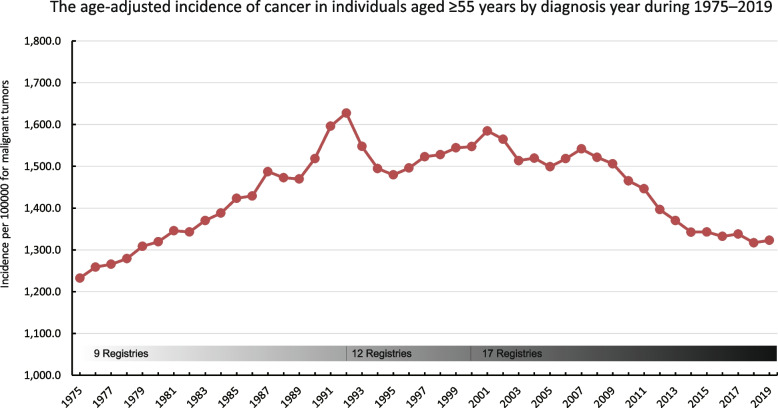
The age-adjusted incidence of cancer in individuals aged ≥ 55 years by diagnosis year during 1975–2019. Data from the SEER database, 1975–1991 data from 9 Registries, 1992–1999 data from 12 Registries, and 2000–2018 data from 17 Registries

Overall, during the period from 1975 to 2019, the age-adjusted incidence rate of cancer in the 55-year-old population followed a three-stage evolution trend: rising, fluctuating, and declining periods. Initially, from 1975 to around 1990, there was an increase in the incidence rate, ranging from 1,200 to 1,500 per 100,000 people, peaking at 1,627.5 per 100,000 people in 1992. Subsequently, between 1993 and 2008, there was a fluctuation period when the incidence rate varied, but remained relatively high, above 1,500 per 100,000 people. From 2008 to 2013, the incidence rate declined to 1,300 per 100,000 people. Finally, between 2014 and 2019, there was a slight slow downward trend, with an incidence rate between 1,350 and 1,300 per 100,000 people (Fig. 1, Supplementary Table 1).

### Annual incidence of cancer in individuals aged ≥  55 years over the past 20 years (2000–2019)

**Fig. 2:**
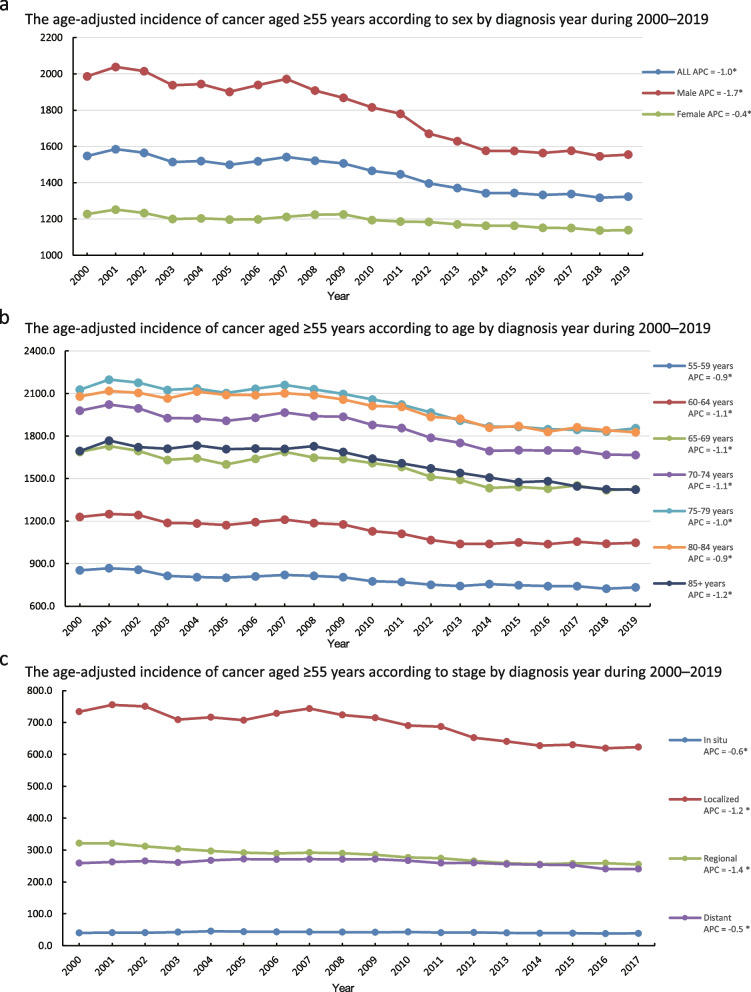
The age-adjusted incidence of cancer in individuals aged ≥ 55 years during 2000 to 2019. **a**, **b** 2000–2019 data from 17 Registries. **a** The age-adjusted incidence of cancer aged ≥ 55 years according to sex by diagnosis year between 2000 and 2019; **b** The age-adjusted incidence of cancer aged ≥ 55 years according to age by diagnosis year between 2000 and 2019; (**c**) The age-adjusted incidence of cancer aged ≥ 55 years according to stage by diagnosis year between 2000 and 2019

The incidence of cancer showed a downward trend over the past 20 years, from 1,547.3 to 1,322.8 per 100,000 population (APC = -1.0; *p* < 0.05). The downward trend in the incidence of cancer in men was greater than that in women, with an APC of -1.7 and -0.4, respectively (*p* < 0.05). However, the overall incidence of cancer in men was higher than that in women (Fig. 2a, Supplementary Table 2.1). Age-stratified analyses revealed that the incidence of cancer is relatively high in individuals aged ≥ 65 years. Specifically, the age groups 65–69, 70–74, 75–79, 80–84, and ≥ 85 all have a high incidence level. Among these groups, the highest incidence is observed in the 75–79 and 80–84 age groups, with almost the same incidence rate (18,300 and 1,860 per 100,000 population). In other words, the incidence rates of cancer in the 75–79 and 80–84 years age groups were the highest. The confidence intervals overlapped when comparing the APCs between the age groups. This implies that although there was a considerable decline in all age groups over time, there was no discernible age-related difference in the trends. However, the results show that the incidence rate in the 70–74 years old group is higher than that in the 65–69 years old group, and this rate in the 75–79 years old group and 80–84 years old group is higher than that in the 70–74 years old group; moreover, the incidence rate of the 85 + group decreased to almost the same as that of the 65–69 years old group (Fig. 2b, Supplementary Table 2.2). In terms of tumour stage, the incidence of cancer was the highest in ‘Localized’, similar in ‘Regional’ and ‘Distant’, and the lowest in ‘In situ’ (Fig. 2c, Supplementary Table 2.3).

### Annual incidence of cancer in individuals aged ≥ 55 years over the past 20 years for different cancer types (2000–2019)

**Fig. 3:**
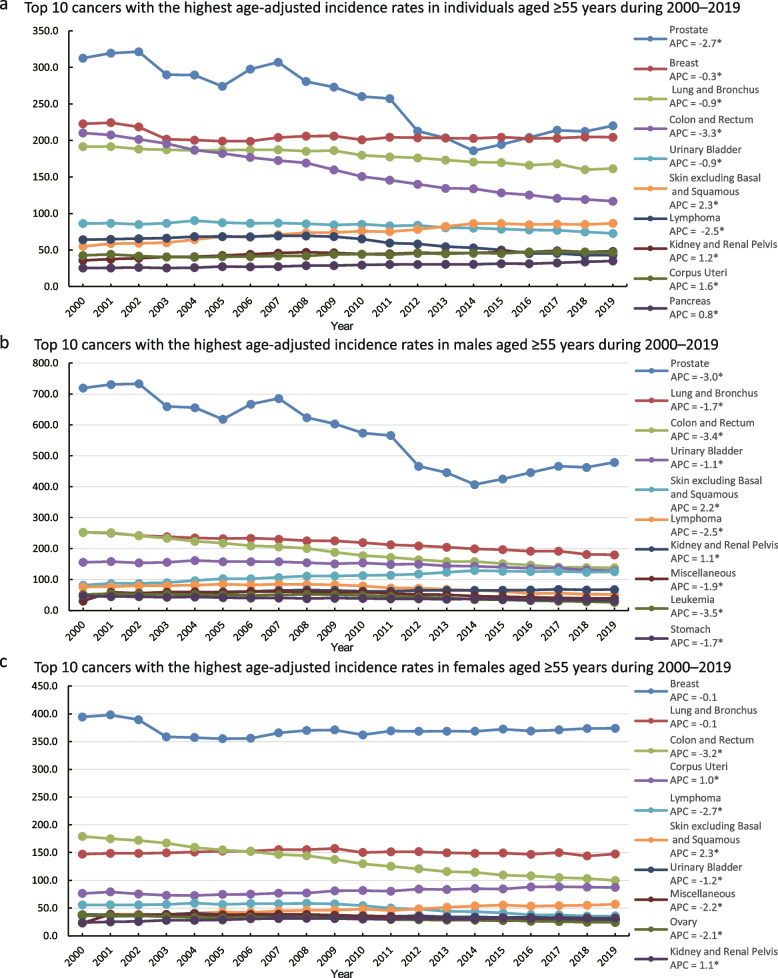
Top 10 cancers with the age-adjusted highest incidence rates (2000–2019) in individuals aged ≥ 55 years. **a**-**c** 2000–2019 data from 17 Registries. **a** Top 10 cancers with the highest age-adjusted incidence rates in individuals aged ≥ 55 years between 2000 and 2019; **b** Top 10 cancers with the highest age-adjusted incidence rates in males aged ≥ 55 years between 2000 and 2019; (**c**) Top 10 cancers with the highest age-adjusted incidence rates in females aged ≥ 55 years between 2000 and 2019

**Fig. 4:**
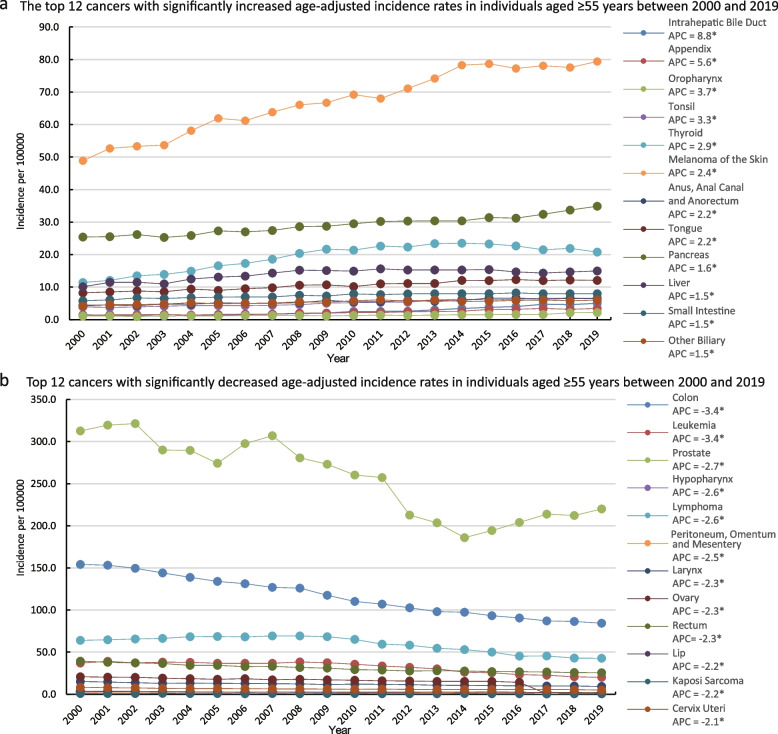
Top 12 cancers with significantly increased or decreased incidence in individuals aged ≥ 55 years. **a**, **b** 2000–2019 data from 17 Registries. **a** The top 12 cancers with significantly increased age-adjusted incidence rates in individuals aged ≥ 55 years between 2000 and 2019; **b** Top 12 cancers with significantly decreased age-adjusted incidence rates in individuals aged ≥ 55 years between 2000 and 2019

The cancers with the highest incidence in men and women were prostate cancer and breast cancer, respectively, followed by lung and bronchial cancer, and colon and rectal cancers (Fig. 3a, b, and c, Supplementary Table 3.1–3.3). Overall, the incidence of prostate cancer showed a downward trend over the past 20 years; however, it showed a slowly increasing trend starting from 2014 (Fig. 3A and B, Supplementary Table 3.1 and 3.2). The incidence rate of breast cancer did not change significantly, and the trend remained relatively flat (APC = 0.1; *p* > 0.05) (Fig. 3C, Supplementary Table 3.3). In women, the incidence of cancer of the corpus uteri showed a slight upward trend (APC = 1.0; *p* < 0.05), whereas that of ovarian cancer showed a decreasing trend (APC = -2.1; *p* < 0.05) (Fig. 3c, Supplementary Table 3.3). Men were more likely to develop urinary tract tumours than women. In addition, men showed a higher incidence of urinary bladder cancer than women. (Fig. 3B and C, Supplementary Table 3.2 and 3.3). The incidence of hepatobiliary system tumours increased significantly over the past 20 years (APC = 8.8; *p* < 0.05). The incidence of intrahepatic cholangiocarcinoma showed the fastest growth despite being low. The incidence rate for some endocrine system tumours, such as pancreatic and thyroid cancers, continued to increase over the past 20 years (APC = 1.6 [*p* < 0.05] and APC = 2.9 [*p* < 0.05], respectively) (Fig. 4a). The incidences of colon and rectal cancer showed a downward trend, with that of colon cancer being more significant (APC = -3.4; *p* < 0.05) (Fig. 4b). There was an increase in the incidences of gastrointestinal cancers affecting the appendix, anus, and small intestine (Fig. 4a), whereas those of leukaemia and lymphoma of the system blood cancers decreased. The incidence of melanoma of the skin, which was high, showed an increasing trend (Fig. 4a).

### Survival rate of cancer in individuals aged ≥ 55 years over the past 20 years

**Fig. 5:**
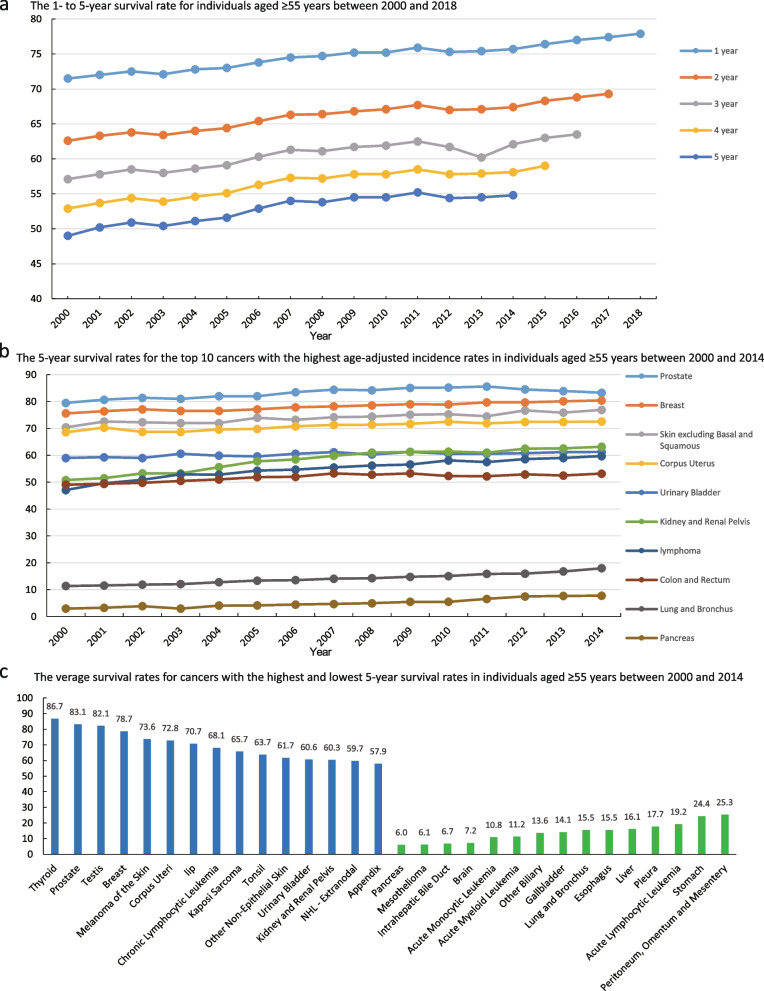
Survival rate of cancer in individuals aged ≥ 55 years. a**-**c 2000–2018data from 17 registries. **a** The 1- to 5-year survival rate for individuals aged ≥ 55 years between 2000 and 2018; **b** The 5-year survival rates for the top 10 cancers with the highest age-adjusted incidence rates in individuals aged ≥ 55 years between 2000 and 2014; (**c**) The average survival rates for cancers with the highest and lowest 5-year survival rates in individuals aged ≥ 55 years between 2000 and 2014

The overall cancer survival rate for individuals aged ≥ 55 years exhibited a steady increase from 2000 to 2019 (Fig. 5a, Supplementary Table 4.1). The overall five-year survival rate in 2000 was approximately 49%, whereas that in 2014 was approximately 55% (Fig. 5A, Supplementary Table 4.1). Among the cancers with high incidence rates from 2000 to 2019, lymphomas and cancers of the kidney and renal pelvis had significantly increased survival rates. Over the past 20 years, the survival rates for breast cancer, skin cancers (excluding basal and squamous), and corpus uteri cancers either remained stable or showed slight improvements. Despite low overall survival rates, lung and bronchial cancers, as well as pancreatic cancer, showed an increasing trend in five-year survival rates, with recent rates of 18% and 7.8%, respectively. The mean five-year survival rate for breast cancer and colon cancer was 78.7% and 51.9%, respectively (Fig. 5B, Supplementary Table 4.2.1–4.2.2). From 2000 to 2019, thyroid and prostate cancers had the highest survival rates, while pancreatic cancer, mesothelioma, and intrahepatic bile duct cancer had the lowest survival rates. The overall five-year survival rates for biliary tract cancers and respiratory system cancers were low (Fig. 5c).

## Discussion

According to the World Health Organization, it is estimated that by 2050, the proportion of the global population aged 60 years or older will increase from 12 to 22%, resulting in a total older population exceeding 2 billion [7]. The incidence of cancer increases considerably with age, and it has been established as the leading cause of death in men and women aged 60–79 years [18]. In 2020, 19.3 million new cases of cancer were recorded worldwide, and the incidence of cancer in high Human Development Index (HDI) countries was higher than that in low HDI regions [2]. However, the incidence of cancer in the United States, a high HDI country, has decreased over the last 20 years, with the incidence rates recorded in recent years similar to those recorded in the 1970s [3]. The results of the present study indicated that the incidence of cancer in individuals was approximately 380 per 100,000 individuals in 2019, slightly higher than the incidence rate of approximately 360 per 100,000 observed in the 1970s. Given that middle and aged individuals are the main cancer population in the United States, the cancer incidence trends in individuals aged ≥ 55 years are consistent with the overall cancer incidence trends, peaking in 1992 and subsequently showing a slowly decreasing trend, especially over the last 20 years (Figs. 1 and 2a).

The reasons for the decline in cancer incidence in the ≥ 85 population are complex. First, the ≥ 85 population had a poor willingness to accept physical examination and medical treatment, resulting in a relatively low rate of tumour examination [19]. Second, the ≥ 85 population has a lower incidence of primary tumours, which may be related to innate genes, including tumour genes and immune system-related genes. These individuals are not susceptible to tumour diseases and therefore live longer [20]. Third, the ≥ 85 population during the years 2000–2019 was at the 1980–1999 stage 20 years ago, which is precisely the stage of the advancement of examination technology and an increase in public awareness of physical examination. The ≥ 85 population underwent tumour screening, which may have resulted in the removal of precancerous diseases [21]. Lastly, among very old people, body functions gradually decline due to ageing; the functioning of cells is also affected, and there is a reduction in body nutrients and growth hormone content. In this environment, the division of cancer cells, or potential cancer cells, slows down. It becomes difficult to grow a sufficiently large tumour, and it is difficult to accumulate more mutations to further metastasise and deteriorate [22, 23].

As a whole, the incidence of each tumour grade showed a downward trend in the past 20 years, as well as the overall incidence. The incidence of carcinoma in situ was relatively low, both because of the difficulty of individual and examination detection of carcinoma in situ and because of the small size of the tumour; thus, the early tumour equivalent to carcinoma in situ was often observed dynamically, without biopsy or definitive diagnosis. The relatively low incidence of ‘Regional’ and ‘Distant’ tumours indicates that the diagnosis, treatment, and screening of cancer are relatively effective in the United States. In many countries and regions, the incidence of ‘Regional’ and ‘Distant’ tumours is relatively high [24]. That in other words, the tumour was identified too late. This reduction in the incidence of cancer in the United States over the past two decades is due to the fact that most patients with cancer are treated at a relatively early stage.

Prostate, colorectal, and lung and bronchial cancers accounted for 17.69%, 10.63%, and 12.07% of the overall incidence from 2000 to 2019, respectively (Supplementary Table 3.1), and played significant roles in contributing to the reduction of cancer incidence. From 2000 to 2019, the incidence of prostate cancer in individuals aged ≥ 55 years decreased by 37.6% (from 709.6 per 100,000 to 442.3 per 100,000). This was mainly due to the reduction of prostate-specific antigen screening for prostate cancer as recommended by the United States Preventive Services Task Force (USPSTF) [25, 26], which recommended against PSA-based screening for men aged 75 years or older in 2008, and against PSA-based prostate cancer screening for all men in 2012. However, the increase in the incidence rate of prostate cancer, which started in 2014, was driven by annual growth in long-term regional-stage and distant-stage diagnoses from 2011 [27]. The current screening process for prostate cancer was established mainly to restore early detection of prostate cancer and reduce over-diagnosis and over-treatment. In 2018, the USPSTF upgraded the screening process to informed decision-making screening for men aged 55–69 years [28, 29]. Regarding colorectal cancer, the incidence in the United States has continued to decline in recent decades [30]. The present study observed a 15.8% reduction in the incidence of colorectal cancer in individuals aged ≥ 55 years over 20 years, declining from 191.6 per 100,000 individuals in 2000 to 161.4 per 100,000 in 2019. This was mainly due to the higher rates of colorectal cancer screening and removal of precancerous polyps, particularly during colonoscopy, in older adults [30–33]. Notably, the incidence of colon cancer in younger patients has increased in recent years; however, their willingness to be screened is lower than that of older patients [34–36]. The change in the incidence rate of lung and bronchial cancer can primarily be attributed to variations in smoking patterns among the population, including tobacco types, smoking habits, and smoking cessation [37]. The smoking rate in the United States reduced from approximately 20% in 2005 to 14% in 2019 [38]. Consequently, the incidence of lung and bronchial cancer in older people has been declining over the past 20 years, significantly more in men than in women, with the latter quitting smoking later and slower [27] (Figs. 3A, B, and C). Screening also plays an important role in the incidence of lung and bronchial cancer. Although screening increased the incidence of stage I disease in patients with lung and bronchial cancer who are older than 65 years, it also significantly decreased the incidence of stage IV disease in this group [39].

In Fig. 3A and Table S3.1, the incidence of breast cancer shows a gentle downward trend in the overall population due to the large base of the denominator, but there is no obvious decline in the female population calculated separately (Fig. 3C). This means that for women, although breast cancer remains at a relatively high level, the incidence of breast cancer has decreased slightly in the last 20 years without significance, indicating that the overall social and female attention to the detection and prevention of breast cancer has remained at a relatively high level in the United States. As seen in Fig. 3C, the decline in breast cancer incidence occurred after 2002, which may be related to the decline in the use of menopausal hormone therapy after the publication of the results of the Women's Health Initiative randomised trial in 2002 [40–42].

The results of the present study revealed an increase in the incidence of hepatobiliary pancreatic neoplasms, with intrahepatic cholangiocarcinoma showing the highest increase (APC = 8.8 (*p* < 0.05) (Fig. 4A). In addition to the effects of chronic inflammation and hepatitis virus infection, obesity and poor lifestyle habits, including smoking, alcohol consumption, and an unhealthy diet, have significantly contributed to the increased incidence of hepatobiliary and pancreatic tumours [43–45].

In the United States, the overall five-year survival rate for all cancers experienced an increase from 49% in the mid-1970s to 68% between 2012 and 2018 [3, 27]. In addition, the survival rates for patients aged ≥ 55 years improved. The data of the present study suggest that the mean 5-year survival rate for all cancers from 2000 to 2014 was 53.7% (Supplementary Table 4.1). The improvement in survival rate can be attributed to early and accurate diagnoses, standardised treatments, multidisciplinary team consultations, strict and standardised training of doctors, and increased focus on the quality of life of patients through modification of therapeutic regimens to improve the physical symptoms, psychological symptoms, and general conditions of patients. In recent decades, the development and progress of targeted therapy and immunotherapy have provided more effective cancer treatment options besides surgery, radiotherapy, and chemotherapy [46–48]. For instance, kidney cancer and lymphoma showed significant increases in survival in our study. Many new immunotherapy drugs significantly improve the prognosis of kidney cancer, which has considerably elevated survival rates [49, 50]. A meta-analysis indicated that lenvatinib combined with pembrolizumab is most likely optimal for the overall survival of patients with kidney cancer [51]. Since 1998 when rituximab was added to standard chemotherapy, the survival rates for non-Hodgkin's lymphoma have improved significantly [52]. Significant improvements in treatment outcomes for relapsed and refractory non-Hodgkin’s lymphoma at all stages have been observed due to advancements in stem cell transplantation techniques, new cytotoxic protocols, and the recent discovery and utilisation of targeted therapies, such as the bcl-2 inhibitor venetoclax and the PD-1 inhibitor pembrolizumab” for improved coherence [53].

As the main risk factors for cancer include smoking, alcohol consumption, insufficient physical activity, and an unhealthy diet, a decrease in the incidence of cancer could be attributed to improvement in disease prevention awareness, diet, and lifestyle habits [54]. Screening task forces generally promote awareness of the importance of early diagnosis and treatment of cancers with high incidence rates, such as prostate, colon, and lung cancers. As the rates of obesity, chronic viral infection, and alcohol consumption increase, the incidences of related cancers, such as liver, bile duct, and pancreatic cancers, also increase. In the United States and other high HDI countries, the development and implementation of smoking cessation policies over the years have played an important role in the prevention and treatment of cancer [55, 56]. For individuals, it is important to adopt healthy lifestyle habits, including maintaining good physical and mental well-being, and being aware of necessary health information to prevent cancer, particularly in older people. However, relying on individual prevention and treatment alone may not be effective in reducing cancer incidence; the involvement of society as a whole may be more effective [54]. Ebrahim and Davey Smith reviewed the existing evidence on health promotion in high-income countries and concluded that there is little evidence of the success of large and expensive health promotion programmes targeted at individuals in reducing cancer incidence [57]. Therefore, implementing social primary prevention policies that target the pathogenic factors of cancer, such as tobacco smoking and obesity, may be effective in reducing the incidence of cancer, especially in an ageing society. Additionally, comprehensive cancer prevention and control plans should encompass surveillance, primary prevention, early detection, treatment, and palliative care [58].

The limitation of this study is that the SEER database used for this study does not include all data from cancer centres. The data collection process is relatively delayed, and the gathered dates could be less accurate in reflecting the current data. The approach of the SEER database to cancer classification or diagnosis has changed significantly over the past four decades. This evolution has led to potential misclassification in cancer diagnoses. Additionally, it is important to acknowledge that the composition of the US population has also changed during this period, with the Hispanic population experiencing significant growth. This demographic shift could impact cancer incidence and survival rates. However, it should be satisfactory and representative for analysing trends in cancer incidence and survival.

In addition, multiple primary cancers were not included in the analysis of this study. This may have affected the incidence numerically. However, based on the conclusion of our analysis, the incidence of multiple primary cancers should be similar to that of single primary cancer. First, the incidence of multiple primary cancer is much lower than that of single primary cancer [59]. Moreover, the main factors affecting the incidence are early screening and prevention of cancer in society overall. With the increasing awareness and attention to multiple primary cancers, there should be a fluctuating trend over time, and it is possible that the curve may lag behind that of simple primary cancer for some time.

## Conclusions

This study revealed that in the United States, the overall incidence of cancer in patients aged ≥ 55 years showed a downward trend over the last 20 years, falling to 13,228 per 100,000 individuals in 2019. In addition, the overall five-year survival rate increased to approximately 55%. This may be mainly due to increased screening for cancers with high incidence rates, such as prostate, colon, and lung cancers, and improved control of risk factors for cancer, such as smoking. The rapid development of targeted therapy and immunotherapy combined with early tumour detection is an important reason for the improved survival of patients with cancer.

### Supplementary Information

**Additional file 1:Supplementary Table 1.** The age-adjusted incidence of cancer in individuals aged ≥ 55 years by diagnosis year during 1975–2019. **Supplementary Table 2.1.** The age-adjusted incidence of cancer aged ≥ 55 years according to sex by diagnosis year during 2000–2019. **Supplementary Table 2.2.** The age-adjusted incidence of cancer aged ≥ 55 years according to age by diagnosis year during 2000–2019. **Supplementary Table 2.3.** The age-adjusted incidence of cancer aged ≥ 55 years according to stage by diagnosis year during 2000–2019. **Supplementary Table 3.1.** Top 10 cancers with the highest age-adjusted incidence rates in individuals aged ≥ 55 years during 2000–2019**.Supplementary Table 3.2.** Top 10 cancers with the highest age-adjusted incidence rates in males aged ≥ 55 years during 2000–2019**.Supplementary Table 3.3.** Top 10 cancers with the highest age-adjusted incidence rates in females aged ≥ 55 years during 2000–2019. **Supplementary Table 4.1.** The 1- to 5-year survival rate for individuals aged ≥ 55 years between 2000 and 2018**.Supplementary Table 4.2.1.** The 5- years survival rates for the top 1–5 cancers with the highest incidence rates in patients older than 55 years (2000–2014). **Supplementary Table 4.2.2.** The 5- years survival rates for the top 6–10 cancers with the highest incidence rates in patients older than 55 years (2000–2014). **Supplementary Table 5.** Site Recode ICD-O-3/WHO 2008 Definition.

## Data Availability

All data generated or analysed during this study are included in this published article and its supplementary information files.

## Abbreviations

SEER: Surveillance, Epidemiology, and End Results
HDI: Human Development Index
APC: Annual percentage change
USPSTF: United States Preventive Services Task Force
ICD-O-3/WHO 2008: International Classification of Disease for Oncology 3rd edition/WHO 2008
