# Supplementary material for: Trends in the incidence and survival of cancer in individuals aged 55 years and older in the United States, 1975–2019
Source: BMC Public Health. 2024 Jan 3;24:72. doi: 10.1186/s12889-023-17571-x (PMC10763484; doi:10.1186/s12889-023-17571-x)
Supplement: Supplementary file 1 — Additional file 1:Supplementary Table 1. The age-adjusted incidence of cancer in individuals aged ≥ 55 years by diagnosis year during 1975–2019. Supplementary Table 2.1. The age-adjusted incidence of cancer aged ≥ 55 years according to sex by diagnosis year during 2000–2019. Supplementary Table 2.2. The age-adjusted incidence of cancer aged ≥ 55 years according to age by diagnosis year during 2000–2019. Supplementary Table 2.3. The age-adjusted incidence of cancer aged ≥ 55 years according to stage by diagnosis year during 2000–2019. Supplementary Table 3.1. Top 10 cancers with the highest age-adjusted incidence rates in individuals aged ≥ 55 years during 2000–2019.Supplementary Table 3.2. Top 10 cancers with the highest age-adjusted incidence rates in males aged ≥ 55 years during 2000–2019.Supplementary Table 3.3. Top 10 cancers with the highest age-adjusted incidence rates in females aged ≥ 55 years during 2000–2019. Supplementary Table 4.1. The 1- to 5-year survival rate for individuals aged ≥ 55 years between 2000 and 2018.Supplementary Table 4.2.1. The 5- years survival rates for the top 1–5 cancers with the highest incidence rates in patients older than 55 years (2000–2014). Supplementary Table 4.2.2. The 5- years survival rates for the top 6–10 cancers with the highest incidence rates in patients older than 55 years (2000–2014). Supplementary Table 5. Site Recode ICD-O-3/WHO 2008 Definition. [file 12889_2023_17571_MOESM1_ESM.docx]

**Supplementary materials**

**Supplementary Table 1. The age-adjusted incidence of cancer in individuals aged≥55 years by diagnosis year during 1975–2019**

| **Registry** | **Year** | **Number of cases**  **(6,847,425)** | **Rate** | 95%CI | |
| --- | --- | --- | --- | --- | --- |
|  |  |  |  | Upper | Lower |
| SEER 9 | 1975 | 36,191 | 1,232.7 | 1,245.8 | 1,219.8 |
|  | 1976 | 37,649 | 1,259.0 | 1,272.0 | 1,246.0 |
|  | 1977 | 38,719 | 1,265.6 | 1,278.5 | 1,252.7 |
|  | 1978 | 40,078 | 1,279.0 | 1,291.8 | 1,266.2 |
|  | 1979 | 41,974 | 1,308.6 | 1,321.4 | 1,295.9 |
|  | 1980 | 43,402 | 1,319.5 | 1,332.2 | 1,306.9 |
|  | 1981 | 45,061 | 1,345.7 | 1,358.4 | 1,333.1 |
|  | 1982 | 45,866 | 1,343.1 | 1,355.7 | 1,330.7 |
|  | 1983 | 47,696 | 1,370.3 | 1,382.8 | 1,357.8 |
|  | 1984 | 49,161 | 1,388.1 | 1,400.6 | 1,375.7 |
|  | 1985 | 51,306 | 1,423.6 | 1,436.1 | 1,411.1 |
|  | 1986 | 52,277 | 1,429.1 | 1,441.5 | 1,416.7 |
|  | 1987 | 55,109 | 1,487.4 | 1,500.0 | 1,474.9 |
|  | 1988 | 55,331 | 1,472.9 | 1,485.3 | 1,460.5 |
|  | 1989 | 55,866 | 1,469.7 | 1,482.1 | 1,457.5 |
|  | 1990 | 58,581 | 1,518.7 | 1,531.1 | 1,506.3 |
|  | 1991 | 62,624 | 1,596.3 | 1,608.9 | 1,583.7 |
| SEER 12 | 1992 | 95,294 | 1,627.5 | 1,617.2 | 1,637.9 |
|  | 1993 | 91,834 | 1,547.8 | 1,537.8 | 1,557.9 |
|  | 1994 | 89,582 | 1,494.8 | 1,485.0 | 1,504.7 |
|  | 1995 | 89,750 | 1,479.7 | 1,470.0 | 1,489.4 |
|  | 1996 | 91,783 | 1,496.0 | 1,486.3 | 1,505.7 |
|  | 1997 | 94,627 | 1,523.1 | 1,513.5 | 1,532.9 |
|  | 1998 | 96,423 | 1,527.9 | 1,518.3 | 1,537.6 |
|  | 1999 | 99,069 | 1,544.3 | 1,534.7 | 1,553.9 |
| SEER 17 | 2000 | 224,105 | 1,547.3 | 1,553.7 | 1,540.9 |
|  | 2001 | 232,939 | 1,584.7 | 1,591.2 | 1,578.3 |
|  | 2002 | 235,379 | 1,564.8 | 1,571.1 | 1,558.5 |
|  | 2003 | 232,755 | 1,513.7 | 1,519.9 | 1,507.5 |
|  | 2004 | 238,157 | 1,519.7 | 1,525.8 | 1,513.6 |
|  | 2005 | 239,928 | 1,499.1 | 1,505.2 | 1,493.1 |
|  | 2006 | 247,929 | 1,518.4 | 1,524.4 | 1,512.3 |
|  | 2007 | 258,324 | 1,541.7 | 1,547.7 | 1,535.7 |
|  | 2008 | 262,148 | 1,521.7 | 1,527.6 | 1,515.8 |
|  | 2009 | 266,976 | 1,506.2 | 1,512.0 | 1,500.4 |
|  | 2010 | 267,133 | 1,465.4 | 1,471.0 | 1,459.7 |
|  | 2011 | 271,084 | 1,446.2 | 1,451.8 | 1,440.7 |
|  | 2012 | 270,114 | 1,396.5 | 1,401.8 | 1,391.1 |
|  | 2013 | 273,200 | 1,370.4 | 1,375.6 | 1,365.1 |
|  | 2014 | 276,140 | 1,342.6 | 1,347.7 | 1,337.5 |
|  | 2015 | 284,445 | 1,343.1 | 1,348.2 | 1,338.1 |
|  | 2016 | 289,929 | 1,332.4 | 1,337.3 | 1,327.4 |
|  | 2017 | 299,252 | 1,338.1 | 1,343.0 | 1,333.2 |
|  | 2018 | 301,818 | 1,317.2 | 1,322.0 | 1,312.4 |
|  | 2019 | 310,417 | 1,322.8 | 1,327.6 | 1,318.1 |

Rates are per 100,000 and age-adjusted to the 2000 US Std Population (19 age groups - Census P25-1130) standard. CI, Confidence intervals.

**Supplementary Table 2.1 The age-adjusted incidence of cancer aged≥55 years according to sex by diagnosis year during 2000–2019**

| **Registry** | **Year** | **Number of cases**  **(5,282,172)** | **Annual age-adjusted incidence rate** | | | | | | | | |
| --- | --- | --- | --- | --- | --- | --- | --- | --- | --- | --- | --- |
|  |  |  | **All age groups** | | | **Males**  **(2,859,999) 54.14%** | | | **Females**  **(2,422,173) 45.86%** | | |
|  |  |  | Rate | 95%CI | | Rate | 95%CI | | Rate | 95%CI | |
|  |  |  |  | Upper | Lower |  | Upper | Lower |  | Upper | Lower |
| SEER 17 | 2000 | 224,105 | 1,547.3 | 1,553.7 | 1,540.9 | 1,986.5 | 1,997.8 | 1,975.3 | 1,227.4 | 1,235.0 | 1,219.8 |
|  | 2001 | 232,939 | 1,584.7 | 1,591.2 | 1,578.3 | 2,038.1 | 2,049.4 | 2,026.8 | 1,252.0 | 1,259.6 | 1,244.4 |
|  | 2002 | 235,379 | 1,564.8 | 1,571.1 | 1,558.5 | 2,014.7 | 2,025.8 | 2,003.6 | 1,232.9 | 1,240.4 | 1,225.4 |
|  | 2003 | 232,755 | 1,513.7 | 1,519.9 | 1,507.5 | 1,937.1 | 1,947.9 | 1,926.4 | 1,199.6 | 1,206.9 | 1,192.3 |
|  | 2004 | 238,157 | 1,519.7 | 1,525.8 | 1,513.6 | 1,943.4 | 1,954.1 | 1,932.7 | 1,203.3 | 1,210.6 | 1,196.1 |
|  | 2005 | 239,928 | 1,499.1 | 1,505.2 | 1,493.1 | 1,900.8 | 1,911.3 | 1,890.4 | 1,196.9 | 1,204.1 | 1,189.7 |
|  | 2006 | 247,929 | 1,518.4 | 1,524.4 | 1,512.3 | 1,938.5 | 1,948.9 | 1,928.0 | 1,198.3 | 1,205.4 | 1,191.1 |
|  | 2007 | 258,324 | 1,541.7 | 1,547.7 | 1,535.7 | 1,971.3 | 1,981.7 | 1,961.0 | 1,212.1 | 1,219.3 | 1,205.0 |
|  | 2008 | 262,148 | 1,521.7 | 1,527.6 | 1,515.8 | 1,907.6 | 1,917.7 | 1,897.5 | 1,224.1 | 1,231.2 | 1,217.1 |
|  | 2009 | 266,976 | 1,506.2 | 1,512.0 | 1,500.4 | 1,868.0 | 1,877.8 | 1,858.2 | 1,225.8 | 1,232.8 | 1,218.8 |
|  | 2010 | 267,133 | 1,465.4 | 1,471.0 | 1,459.7 | 1,815.1 | 1,824.7 | 1,805.6 | 1,193.9 | 1,200.7 | 1,187.1 |
|  | 2011 | 271,084 | 1,446.2 | 1,451.8 | 1,440.7 | 1,779.8 | 1,789.1 | 1,770.4 | 1,185.9 | 1,192.7 | 1,179.2 |
|  | 2012 | 270,114 | 1,396.5 | 1,401.8 | 1,391.1 | 1,670.4 | 1,679.4 | 1,661.6 | 1,184.0 | 1,190.7 | 1,177.4 |
|  | 2013 | 273,200 | 1,370.4 | 1,375.6 | 1,365.1 | 1,629.2 | 1,637.9 | 1,620.6 | 1,170.3 | 1,176.8 | 1,163.8 |
|  | 2014 | 276,140 | 1,342.6 | 1,347.7 | 1,337.5 | 1,576.1 | 1,584.4 | 1,567.7 | 1,162.7 | 1,169.0 | 1,156.3 |
|  | 2015 | 284,445 | 1,343.1 | 1,348.2 | 1,338.1 | 1,574.8 | 1,583.1 | 1,566.6 | 1,163.2 | 1,169.5 | 1,156.9 |
|  | 2016 | 289,929 | 1,332.4 | 1,337.3 | 1,327.4 | 1,563.9 | 1,572.0 | 1,555.8 | 1,151.3 | 1,157.5 | 1,145.1 |
|  | 2017 | 299,252 | 1,338.1 | 1,343.0 | 1,333.2 | 1,576.6 | 1,584.6 | 1,568.6 | 1,150.0 | 1,156.1 | 1,144.0 |
|  | 2018 | 301,818 | 1,317.2 | 1,322.0 | 1,312.4 | 1,545.8 | 1,553.6 | 1,538.0 | 1,136.3 | 1,142.3 | 1,130.4 |
|  | 2019 | 310,417 | 1,322.8 | 1,327.6 | 1,318.1 | 1,554.9 | 1,562.6 | 1,547.3 | 1,138.6 | 1,144.5 | 1,132.7 |
|  | 2000-2019 | 5282172 | 1,436.2 | 1,437.4 | 1,434.9 | 1761.2 | 1,763.3 | 1,759.2 | 1186.3 | 1,187.8 | 1,184.8 |
| APC  95%CI | - | - | -1.0*  -0.9, -1.2 | | | -1.7*  -1,4, -1,9 | | | -0.4*  -0.3, -0.5 | | |

Rates are per 100,000 and age-adjusted to the 2000 US Std Population (19 age groups - Census P25-1130) standard.

APC, Annual percentage change. CI, Confidence intervals. *, p < 0.05.

**Supplementary Table 2.2 The age-adjusted incidence of cancer aged≥55 years according to age by diagnosis year during 2000–2019**

| **Registry** | **Year** | **Annual age-adjusted incidence rate** | | | | | | | | | | | | | | | | | | | | |
| --- | --- | --- | --- | --- | --- | --- | --- | --- | --- | --- | --- | --- | --- | --- | --- | --- | --- | --- | --- | --- | --- | --- |
|  |  | **55–59 years old**  **(763,325) 14.45%** | | | **60–64 years old**  **(900,309) 17.04%** | | | **65–69 years old**  **(982,965) 18.61%** | | | **70–74 years old**  **(896,578)16.97%** | | | **75–79 years old**  **(761,097) 14.41%** | | | **80–84 years old**  **(550,157) 10.41%** | | | **85+ years old**  **(427,741)8.10%** | | |
|  |  | Rate | 95%CI | | Rate | 95%CI | | Rate | 95%CI | | Rate | 95%CI | | Rate | 95%CI | | Rate | 95%CI | | Rate | 95%CI | |
|  |  |  | Upper | Lower |  | Upper | Lower |  | Upper | Lower |  | Upper | Lower |  | Upper | Lower |  | Upper | Lower |  | Upper | Lower |
| SEER 17 | 2000 | 852.9 | 862.7 | 843.2 | 1,228.8 | 1,242.1 | 1,215.6 | 1,688.5 | 1,705.3 | 1,671.8 | 1,979.1 | 1,998.1 | 1,960.2 | 2,127.5 | 2,148.9 | 2,106.3 | 2,079.6 | 2,105.6 | 2,053.9 | 1,694.5 | 1,720.0 | 1,669.4 |
|  | 2001 | 867.5 | 877.2 | 857.9 | 1,249.8 | 1,263.0 | 1,236.7 | 1,728.6 | 1,745.6 | 1,711.7 | 2,022.4 | 2,041.7 | 2,003.3 | 2,198.2 | 2,220.0 | 2,176.7 | 2,117.6 | 2,143.3 | 2,092.1 | 1,768 | 1,793.8 | 1,742.5 |
|  | 2002 | 857.4 | 866.7 | 848.2 | 1,243.1 | 1,256.1 | 1,230.3 | 1,695.2 | 1,712.0 | 1,678.6 | 1,996 | 2,015.2 | 1,977.0 | 2,176.2 | 2,197.8 | 2,154.7 | 2,105 | 2,130.2 | 2,080.1 | 1,722.1 | 1,747.3 | 1,697.2 |
|  | 2003 | 813.8 | 822.6 | 805.1 | 1,187 | 1,199.3 | 1,174.7 | 1,631.7 | 1,647.9 | 1,615.6 | 1,926.9 | 1,945.9 | 1,908.2 | 2,125.5 | 2,146.9 | 2,104.3 | 2,065.3 | 2,090.0 | 2,040.9 | 1,710.7 | 1,735.5 | 1,686.1 |
|  | 2004 | 805 | 813.5 | 796.5 | 1,184.1 | 1,196.2 | 1,172.2 | 1,643.7 | 1,659.8 | 1,627.7 | 1,924.4 | 1,943.4 | 1,905.6 | 2,135.1 | 2,156.6 | 2,113.8 | 2,113.9 | 2,138.5 | 2,089.5 | 1,734 | 1,758.8 | 1,709.5 |
|  | 2005 | 800.9 | 809.2 | 792.6 | 1,172 | 1,183.8 | 1,160.3 | 1,600 | 1,615.7 | 1,584.4 | 1907.2 | 1,926.1 | 1,888.5 | 2,103.5 | 2,124.8 | 2,082.3 | 2,090.4 | 2,114.8 | 2,066.3 | 1,707.9 | 1,732.0 | 1,684.0 |
|  | 2006 | 809.5 | 817.7 | 801.4 | 1,192.3 | 1,204.0 | 1,180.7 | 1,639.6 | 1,655.3 | 1,624.0 | 1,929.7 | 1,948.6 | 1,910.9 | 2,133.1 | 2,154.6 | 2,111.7 | 2,089 | 2,113.3 | 2,064.9 | 1,711.9 | 1,735.6 | 1,688.5 |
|  | 2007 | 820.4 | 828.6 | 812.2 | 1,210.6 | 1,221.9 | 1,199.4 | 1,688.1 | 1,703.7 | 1,672.6 | 1966.3 | 1,985.3 | 1,947.4 | 2160 | 2,181.7 | 2,138.4 | 2,102 | 2,126.3 | 2,077.9 | 1,709 | 1,732.2 | 1,686.0 |
|  | 2008 | 813.4 | 821.5 | 805.4 | 1,185.7 | 1,196.7 | 1,174.9 | 1,647.5 | 1,662.5 | 1,632.6 | 1,940 | 1,958.7 | 1,921.5 | 2,130 | 2,151.6 | 2,108.5 | 2,088.4 | 2,112.6 | 2,064.4 | 1,728.8 | 1,751.8 | 1,706.2 |
|  | 2009 | 804.1 | 812.0 | 796.2 | 1,176.7 | 1,187.3 | 1,166.2 | 1,638.3 | 1,652.9 | 1,623.8 | 1,936.5 | 1,954.9 | 1,918.2 | 2097.2 | 2,118.6 | 2,075.9 | 2,057.6 | 2,081.6 | 2,033.8 | 1,687.3 | 1,709.6 | 1,665.3 |
|  | 2010 | 775.5 | 783.2 | 767.8 | 1,128.7 | 1,138.8 | 1,118.7 | 1,609.1 | 1,623.3 | 1,595.0 | 1,878.1 | 1,896.1 | 1,860.3 | 2057.5 | 2,078.7 | 2,036.4 | 2,012.3 | 2,036.0 | 1,988.8 | 1,640.1 | 1,661.7 | 1,618.8 |
|  | 2011 | 770.2 | 777.7 | 762.6 | 1,110.8 | 1,120.6 | 1,101.1 | 1,582.5 | 1,596.3 | 1,568.7 | 1,857.1 | 1,874.7 | 1,839.7 | 2021.8 | 2,042.8 | 2,001.1 | 2,006.4 | 2,030.0 | 1,983.0 | 1,608.3 | 1,629.3 | 1,587.5 |
|  | 2012 | 750.6 | 758.0 | 743.3 | 1,066.2 | 1,075.8 | 1,056.7 | 1,512.6 | 1,525.5 | 1,499.6 | 1,787.4 | 1,804.3 | 1,770.7 | 1,966 | 1,986.5 | 1,945.7 | 1,935.2 | 1,958.3 | 1,912.2 | 1,571.8 | 1,592.3 | 1,551.5 |
|  | 2013 | 742.4 | 749.7 | 735.2 | 1,039.7 | 1,049.1 | 1,030.4 | 1,490.8 | 1,503.4 | 1,478.2 | 1,751.6 | 1,767.8 | 1,735.4 | 1909.2 | 1,929.2 | 1,889.5 | 1,922.1 | 1,945.2 | 1,899.2 | 1,539.6 | 1,559.6 | 1,519.7 |
|  | 2014 | 755.7 | 762.9 | 748.5 | 1,038.8 | 1,048.1 | 1,029.7 | 1433.3 | 1,445.3 | 1,421.3 | 1,694.7 | 1,710.3 | 1,679.2 | 1,867 | 1,886.4 | 1,847.7 | 1,859 | 1,881.7 | 1,836.5 | 1,507.7 | 1,527.3 | 1,488.2 |
|  | 2015 | 747.2 | 754.3 | 740.0 | 1,050.2 | 1,059.4 | 1,041.1 | 1,441.5 | 1,453.3 | 1,429.7 | 1,700 | 1,715.3 | 1,684.8 | 1,864.7 | 1,883.8 | 1,845.7 | 1,871.1 | 1,893.8 | 1,848.7 | 1,474.2 | 1,493.4 | 1,455.2 |
|  | 2016 | 741.7 | 748.8 | 734.6 | 1,037.7 | 1,046.8 | 1,028.8 | 1,428.5 | 1,440.0 | 1,417.0 | 1,698.6 | 1,713.7 | 1,683.7 | 1,848.5 | 1,867.3 | 1,829.9 | 1829.7 | 1,852.0 | 1,807.6 | 1,481.7 | 1,500.7 | 1,462.9 |
|  | 2017 | 740.5 | 747.6 | 733.4 | 1,054.8 | 1,063.8 | 1,045.9 | 1,451.7 | 1,463.3 | 1,440.2 | 1,696.9 | 1,711.3 | 1,682.6 | 1,844.2 | 1,862.5 | 1,826.0 | 1861.3 | 1,883.6 | 1,839.2 | 1,444.7 | 1,463.4 | 1,426.2 |
|  | 2018 | 723.0 | 730.1 | 716.1 | 1,040.2 | 1,049.0 | 1,031.4 | 1,418.1 | 1,429.5 | 1,406.8 | 1,668.1 | 1,682.1 | 1,654.2 | 1,832.9 | 1,850.7 | 1,815.2 | 1840.6 | 1,862.5 | 1,819.0 | 1,424.6 | 1,443.0 | 1,406.4 |
|  | 2019 | 732.5 | 739.6 | 725.5 | 1,047.2 | 1,056.0 | 1,038.4 | 1,425 | 1,436.3 | 1,413.8 | 1,666.5 | 1,680.1 | 1,652.9 | 1,854.7 | 1,872.2 | 1,837.3 | 1826.4 | 1,847.8 | 1,805.1 | 1,421.9 | 1,440.2 | 1,403.8 |
|  | 2000-2019 | 780.2 | 782.0 | 778.5 | 1,117.3 | 1,119.6 | 1,115.0 | 1,547 | 1,550.0 | 1,543.9 | 1,826.7 | 1,830.4 | 1,822.9 | 2,013.6 | 2,018.1 | 2,009.1 | 1,989.1 | 1,994.4 | 1,983.9 | 1,597.0 | 1,601.8 | 1,592.2 |
| APC  95%CI | - | -0.9*  -0.8, -1.0 | | | -1.1*  -0.9, -1.3 | | | -1.1*  -0.9, -1.3 | | | -1.1*  -0.9, -1.3 | | | -1.0*  -0.8, -1.2 | | | -0.9*  -0.7, -1.1 | | | -1.2*  -1.0, -1.4 | | |

Rates are per 100,000 and age-adjusted to the 2000 US Std Population (19 age groups - Census P25-1130) standard.

APC, Annual percentage change. CI, Confidence intervals. *, p < 0.05.

**Supplementary Table 2.3 The age-adjusted incidence of cancer aged≥55 years according to stage by diagnosis year during 2000–2019**

| **Registry** | **Year** | **Number of cases**  **(4,084,649)** | **Annual age-adjusted incidence rate** | | | | | | | | | | | | | | |
| --- | --- | --- | --- | --- | --- | --- | --- | --- | --- | --- | --- | --- | --- | --- | --- | --- | --- |
|  |  |  | **All stage**  **(4,084,649)** | | | **In situ**  **(128,622)**  **3.15%** | | | **Localized**  **(2,216,839)**  **54.27%** | | | **Regional**  **(909,178)**  **22.25%** | | | **Distant**  **(763,325)**  **18.69%** | | |
|  |  |  | Rate | 95%CI | | Rate | 95%CI | | Rate | 95%CI | | Rate | 95%CI | | Rate | 95%CI | |
|  |  |  |  | Upper | Lower |  | Upper | Lower |  | Upper | Lower |  | Upper | Lower |  | Upper | Lower |
| SEER 17 | 2000 | 196,228 | 40.2 | 41.2 | 39.1 | 733.8 | 738.2 | 729.4 | 733.8 | 738.2 | 729.4 | 321.4 | 324.3 | 318.5 | 258.9 | 261.5 | 256.3 |
|  | 2001 | 202,935 | 41.1 | 42.1 | 40.0 | 755.4 | 759.9 | 751.0 | 755.4 | 759.9 | 751.0 | 321.1 | 324.0 | 318.2 | 262.4 | 265.1 | 259.8 |
|  | 2002 | 205,962 | 40.6 | 41.6 | 39.6 | 750.5 | 754.9 | 746.1 | 750.5 | 754.9 | 746.1 | 311.8 | 314.7 | 309.0 | 265.4 | 268.0 | 262.8 |
|  | 2003 | 202,484 | 42.5 | 43.6 | 41.5 | 708.9 | 713.1 | 704.7 | 708.9 | 713.1 | 704.7 | 303.5 | 306.3 | 300.8 | 260.7 | 263.3 | 258.2 |
|  | 2004 | 208,116 | 45.4 | 46.5 | 44.3 | 716.5 | 720.8 | 712.3 | 716.5 | 720.8 | 712.3 | 296.9 | 299.6 | 294.2 | 267.7 | 270.3 | 265.2 |
|  | 2005 | 210,537 | 44.0 | 45.0 | 42.9 | 707.0 | 711.1 | 702.8 | 707.0 | 711.1 | 702.8 | 291.5 | 294.1 | 288.8 | 271.4 | 274.0 | 268.9 |
|  | 2006 | 217,809 | 43.4 | 44.4 | 42.4 | 728.8 | 733.0 | 724.7 | 728.8 | 733.0 | 724.7 | 289.2 | 291.8 | 286.6 | 270.5 | 273.1 | 268.0 |
|  | 2007 | 226,615 | 43.2 | 44.2 | 42.1 | 743.8 | 748.0 | 739.6 | 743.8 | 748.0 | 739.6 | 291.9 | 294.5 | 289.3 | 271.2 | 273.7 | 268.7 |
|  | 2008 | 229,076 | 42.5 | 43.5 | 41.5 | 723.6 | 727.7 | 719.6 | 723.6 | 727.7 | 719.6 | 289.7 | 292.2 | 287.1 | 270.9 | 273.5 | 268.5 |
|  | 2009 | 233,434 | 41.9 | 42.9 | 40.9 | 714.7 | 718.7 | 710.7 | 714.7 | 718.7 | 710.7 | 285.4 | 287.9 | 282.9 | 271.6 | 274.1 | 269.1 |
|  | 2010 | 233,447 | 43.0 | 44.0 | 42.0 | 690.4 | 694.3 | 686.6 | 690.4 | 694.3 | 686.6 | 276.7 | 279.1 | 274.2 | 266.8 | 269.2 | 264.4 |
|  | 2011 | 237,129 | 40.9 | 41.9 | 40.0 | 687.0 | 690.8 | 683.2 | 687.0 | 690.8 | 683.2 | 274.2 | 276.6 | 271.8 | 258.9 | 261.3 | 256.5 |
|  | 2012 | 236,637 | 41.7 | 42.7 | 40.8 | 652.1 | 655.8 | 648.5 | 652.1 | 655.8 | 648.5 | 265.9 | 268.3 | 263.6 | 259.7 | 262.0 | 257.3 |
|  | 2013 | 239,025 | 40.1 | 41.0 | 39.2 | 640.6 | 644.2 | 637.0 | 640.6 | 644.2 | 637.0 | 258.6 | 260.9 | 256.4 | 255.7 | 258.0 | 253.4 |
|  | 2014 | 242,554 | 39.2 | 40.1 | 38.3 | 627.2 | 630.6 | 623.7 | 627.2 | 630.6 | 623.7 | 255.5 | 257.7 | 253.3 | 253.8 | 256.1 | 251.6 |
|  | 2015 | 250,779 | 39.2 | 40.1 | 38.3 | 630.4 | 633.8 | 626.9 | 630.4 | 633.8 | 626.9 | 257.9 | 260.1 | 255.7 | 252.6 | 254.8 | 250.4 |
|  | 2016 | 252,490 | 37.9 | 38.8 | 37.1 | 619.4 | 622.7 | 616.0 | 619.4 | 622.7 | 616.0 | 259.0 | 261.2 | 256.8 | 240.2 | 242.3 | 238.0 |
|  | 2017 | 259,392 | 38.5 | 39.4 | 37.7 | 622.8 | 626.1 | 619.5 | 622.8 | 626.1 | 619.5 | 254.6 | 256.7 | 252.5 | 240.3 | 242.4 | 238.2 |
|  | 2000-2017 | 4,084,649 | 41.2 | 41.4 | 41.0 | 686.4 | 687.3 | 685.5 | 686.4 | 687.3 | 685.5 | 280.7 | 281.2 | 280.1 | 259.7 | 260.3 | 259.2 |
| APC  95%CI | - | - | -1.1*  -0.9,-1.3 | | | -0.6*  -0.9, -1.5 | | | -1.2*  -1.9, -1,4 | | | -1.4*  -1.3, -1.6 | | | -0.5*  -0.2, -0.8 | | |

Rates are per 100,000 and age-adjusted to the 2000 US Std Population (19 age groups - Census P25-1130) standard.

APC, Annual percentage change. CI, Confidence intervals. *, p < 0.05.

**Supplementary Table 3.1. Top 10 cancers with the highest age-adjusted incidence rates in individuals aged≥55 years during 2000–2019**

| **Registry** | **Year** | **Annual age-adjusted incidence rate** | | | | | | | | | | | | | | | | | | | | | | | | | | | | | |
| --- | --- | --- | --- | --- | --- | --- | --- | --- | --- | --- | --- | --- | --- | --- | --- | --- | --- | --- | --- | --- | --- | --- | --- | --- | --- | --- | --- | --- | --- | --- | --- |
|  |  | **Prostate**  **(934,596)**  **17.69%** | | | **Breast**  **(768,013)**  **14.54%** | | | **Lung and Bronchus**  **(637,521)**  **12.07%** | | | **Colon and Rectum**  **(561,547)**  **10.63%** | | | **Urinary Bladder**  **(292,631)**  **5.54%** | | | **Skin excluding Basal and Squamous**  **(276,087)**  **5.23%** | | | **Lymphoma**  **(211,343)**  **4.00%** | | | **Kidney and Renal Pelvis**  **(166,830)**  **3.16%** | | | **Corpus Uteri**  **(168,863)**  **3.12%** | | | **Pancreas**  **(107,031)**  **2.03%** | | |
|  |  | Rate | 95%CI | | Rate | 95%CI | | Rate | 95%CI | | Rate | 95%CI | | Rate | 95%CI | | Rate | 95%CI | | Rate | 95%CI | | Rate | 95%CI | | Rate | 95%CI | | Rate | 95%CI | |
|  |  |  | Upper | Lower |  | Upper | Lower |  | Upper | Lower |  | Upper | Lower |  | Upper | Lower |  | Upper | Lower |  | Upper | Lower |  | Upper | Lower |  | Upper | Lower |  | Upper | Lower |
| SEER 17 | **2000** | **312.6** | **315.4** | **309.7** | **222.8** | **225.2** | **220.3** | **191.6** | **193.9** | **189.4** | **210.2** | **212.6** | **207.9** | **86.2** | **82.6** | **84.7** | **55.0** | **56.3** | **53.8** | **64.1** | **65.4** | **62.8** | **35.8** | **36.8** | **34.8** | **42.6** | **44.6** | **42.4** | **25.4** | **29.6** | **29.3** |
|  | **2001** | **319.4** | **322.4** | **316.6** | **224.4** | **226.9** | **222.0** | **191.7** | **194.0** | **189.5** | **207.5** | **209.9** | **205.2** | **86.7** | **87.8** | **85.2** | **58.8** | **60.0** | **57.5** | **64.7** | **66.0** | **63.4** | **37.5** | **38.5** | **36.5** | **44.0** | **46.0** | **43.8** | **25.5** | **26.2** | **24.6** |
|  | **2002** | **321.4** | **324.3** | **318.6** | **218.5** | **220.9** | **216.2** | **188.2** | **190.4** | **186.0** | **201.5** | **203.8** | **199.2** | **85.1** | **88.3** | **83.6** | **59.3** | **60.6** | **58.1** | **65.5** | **66.9** | **64.2** | **38.7** | **39.7** | **37.7** | **41.8** | **43.5** | **41.4** | **26.2** | **26.3** | **24.7** |
|  | **2003** | **290.0** | **292.7** | **287.3** | **201.6** | **203.8** | **199.3** | **187.2** | **189.4** | **185.0** | **195.7** | **197.9** | **193.5** | **86.7** | **86.6** | **85.2** | **59.9** | **61.1** | **58.7** | **66.4** | **67.7** | **65.1** | **40.6** | **41.6** | **39.6** | **40.3** | **42.1** | **40.1** | **25.3** | **27.0** | **25.4** |
|  | **2004** | **289.5** | **292.2** | **286.8** | **200.5** | **202.7** | **198.3** | **186.4** | **188.6** | **184.3** | **186.9** | **189.1** | **184.8** | **90.2** | **88.2** | **88.7** | **64.2** | **65.5** | **62.9** | **68.4** | **69.7** | **67.1** | **40.9** | **41.9** | **39.9** | **40.0** | **41.7** | **39.7** | **25.9** | **26.1** | **24.5** |
|  | **2005** | **274.1** | **276.7** | **271.6** | **199.0** | **201.2** | **196.9** | **186.6** | **188.7** | **184.4** | **182.3** | **184.4** | **180.2** | **87.4** | **91.7** | **85.9** | **68.2** | **69.5** | **66.9** | **68.5** | **69.8** | **67.2** | **42.5** | **43.5** | **41.5** | **40.9** | **42.7** | **40.7** | **27.3** | **26.8** | **25.1** |
|  | **2006** | **297.5** | **300.2** | **294.8** | **198.9** | **201.0** | **196.7** | **187.1** | **189.2** | **185.0** | **176.8** | **178.9** | **174.7** | **86.5** | **88.9** | **85.0** | **67.7** | **69.0** | **66.4** | **68.2** | **69.5** | **67.0** | **44.2** | **45.2** | **43.2** | **41.0** | **42.7** | **40.7** | **27.0** | **28.1** | **26.5** |
|  | **2007** | **307.0** | **309.6** | **304.3** | **204.1** | **206.3** | **202.0** | **187.2** | **189.3** | **185.1** | **172.5** | **174.5** | **170.4** | **86.9** | **88.0** | **85.4** | **70.9** | **72.2** | **69.6** | **69.3** | **70.6** | **68.1** | **45.8** | **46.9** | **44.8** | **41.9** | **43.6** | **41.6** | **27.4** | **27.8** | **26.2** |
|  | **2008** | **280.7** | **283.3** | **278.2** | **205.9** | **208.1** | **203.8** | **185.2** | **187.3** | **183.1** | **169.2** | **171.2** | **167.3** | **85.9** | **88.3** | **84.5** | **73.6** | **74.9** | **72.3** | **69.3** | **70.6** | **68.0** | **46.8** | **47.8** | **45.7** | **41.9** | **43.7** | **41.8** | **28.6** | **28.2** | **26.6** |
|  | **2009** | **273.0** | **275.5** | **270.6** | **206.1** | **208.2** | **204.0** | **186.1** | **188.2** | **184.1** | **159.8** | **161.7** | **157.9** | **84.4** | **87.3** | **83.1** | **74.0** | **75.3** | **72.8** | **68.4** | **69.7** | **67.2** | **46.2** | **47.2** | **45.2** | **44.0** | **46.0** | **44.1** | **28.7** | **29.5** | **27.8** |
|  | **2010** | **260.2** | **262.6** | **257.9** | **200.8** | **202.9** | **198.7** | **179.8** | **181.8** | **177.8** | **150.6** | **152.4** | **148.8** | **85.3** | **85.9** | **84.0** | **75.8** | **77.1** | **74.5** | **65.1** | **66.3** | **63.9** | **44.4** | **45.3** | **43.4** | **44.2** | **46.1** | **44.2** | **29.5** | **29.5** | **27.9** |
|  | **2011** | **257.3** | **259.7** | **255.0** | **204.3** | **206.4** | **202.3** | **177.5** | **179.5** | **175.5** | **145.7** | **147.5** | **143.9** | **83.0** | **86.7** | **81.7** | **75.0** | **76.3** | **73.8** | **59.5** | **60.6** | **58.3** | **44.8** | **45.8** | **43.8** | **43.4** | **45.5** | **43.6** | **30.2** | **30.3** | **28.6** |
|  | **2012** | **212.7** | **214.8** | **210.6** | **203.6** | **205.6** | **201.5** | **176.2** | **178.1** | **174.2** | **140.1** | **141.8** | **138.4** | **83.9** | **84.4** | **82.6** | **78.3** | **79.6** | **77.0** | **58.4** | **59.5** | **57.3** | **46.2** | **47.1** | **45.2** | **45.4** | **47.5** | **45.6** | **30.3** | **31.0** | **29.4** |
|  | **2013** | **203.5** | **205.5** | **201.5** | **203.3** | **205.3** | **201.3** | **173.2** | **175.1** | **171.3** | **134.5** | **136.2** | **132.9** | **80.5** | **85.3** | **79.2** | **82.3** | **83.6** | **81.0** | **54.6** | **55.7** | **53.6** | **45.8** | **46.8** | **44.9** | **44.9** | **46.8** | **45.0** | **30.4** | **31.1** | **29.5** |
|  | **2014** | **185.9** | **187.8** | **184.1** | **202.7** | **204.7** | **200.7** | **170.4** | **172.3** | **168.6** | **133.9** | **135.5** | **132.3** | **80.0** | **81.8** | **78.8** | **86.3** | **87.6** | **85.0** | **53.0** | **54.0** | **51.9** | **45.8** | **46.7** | **44.9** | **45.8** | **47.8** | **45.9** | **30.4** | **31.2** | **29.6** |
|  | **2015** | **194.4** | **196.3** | **192.6** | **204.5** | **206.5** | **202.6** | **169.7** | **171.5** | **167.9** | **128.2** | **129.7** | **126.6** | **78.7** | **81.3** | **77.5** | **86.3** | **87.6** | **85.0** | **50.0** | **51.0** | **49.0** | **47.1** | **48.1** | **46.2** | **45.3** | **47.5** | **45.6** | **31.4** | **31.1** | **29.6** |
|  | **2016** | **204.0** | **206.0** | **202.1** | **202.6** | **204.5** | **200.7** | **166.1** | **167.9** | **164.4** | **125.4** | **127.0** | **123.9** | **77.5** | **80.0** | **76.3** | **84.8** | **86.1** | **83.5** | **45.2** | **46.1** | **44.3** | **47.1** | **48.0** | **46.2** | **47.3** | **49.4** | **47.6** | **31.2** | **32.2** | **30.6** |
|  | **2017** | **213.9** | **215.8** | **211.9** | **203.1** | **205.0** | **201.3** | **168.1** | **169.8** | **166.3** | **120.7** | **122.2** | **119.2** | **77.0** | **78.8** | **75.9** | **85.5** | **86.8** | **84.3** | **45.4** | **46.3** | **44.5** | **49.0** | **49.9** | **48.1** | **47.5** | **49.6** | **47.7** | **32.4** | **32.0** | **30.5** |
|  | **2018** | **212.3** | **214.2** | **210.4** | **204.7** | **206.6** | **202.9** | **160.0** | **161.7** | **158.3** | **119.2** | **120.7** | **117.8** | **74.7** | **78.3** | **73.5** | **84.9** | **86.1** | **83.6** | **42.9** | **43.8** | **42.0** | **47.3** | **48.2** | **46.4** | **47.0** | **49.1** | **47.3** | **33.7** | **33.2** | **31.7** |
|  | **2019** | **220.1** | **222.0** | **218.3** | **204.5** | **206.3** | **202.7** | **161.4** | **163.1** | **159.7** | **116.9** | **118.3** | **115.5** | **72.8** | **75.8** | **71.7** | **86.5** | **87.7** | **85.3** | **42.8** | **43.7** | **42.0** | **48.2** | **49.1** | **47.3** | **46.6** | **49.0** | **47.2** | **34.9** | **34.5** | **32.9** |
|  | **2000-2019** | **250.5** | **251.0** | **250.0** | **205.5** | **205.9** | **205.0** | **176.8** | **177.2** | **176.3** | **154.8** | **155.2** | **154.4** | **82.2** | **82.6** | **81.9** | **75.2** | **75.5** | **74.9** | **58.2** | **58.4** | **57.9** | **44.7** | **44.9** | **44.5** | **44.3** | **45.5** | **45.0** | **29.4** | **29.6** | **29.3** |
| APC  95%CI | - | **-2.7 ***  **-2.0, -3.3** | | | **-0.3 ***  **-0.0, -0.5** | | | **-0.9 ***  **-0.8, -1.1** | | | **-3.3 ***  **-3.1, -3.4** | | | **-0.9 ***  **-0.7, -1.1** | | | **2.3 ***  **-2.6, -2.0** | | | **-2.5 ***  **-1.8, -3.3** | | | **1.2 ***  **-1.5, -0.9** | | | **0.8 ***  **-1.0, -0.6** | | | **-1.6 ***  **-1.8, -1.5** | | |

Rates are per 100,000 and age-adjusted to the 2000 US Std Population (19 age groups - Census P25-1130) standard.

APC, Annual percentage change. CI, Confidence intervals. *, p < 0.05.

**Supplementary Table 3.2. Top 10 cancers with the highest age-adjusted incidence rates in males aged ≥55 years during 2000–2019**

| **Registry** | **Year** | **Annual age-adjusted incidence rate** | | | | | | | | | | | | | | | | | | | | | | | | | | | | | |
| --- | --- | --- | --- | --- | --- | --- | --- | --- | --- | --- | --- | --- | --- | --- | --- | --- | --- | --- | --- | --- | --- | --- | --- | --- | --- | --- | --- | --- | --- | --- | --- |
|  |  | **Prostate**  **(934,596)**  **32.68%** | | | **Lung and Bronchus**  **(339,618)**  **11.87%** | | | **Colon and Rectum**  **(290,374)**  **11.21%** | | | **Urinary Bladder**  **(223,015)**  **7.80%** | | | **Skin excluding Basal and Squamous**  **(177,671)**  **6.21%** | | | **Lymphoma**  **(112,193)**  **3.92%** | | | **Kidney and Renal Pelvis**  **(106,088)**  **3.71%** | | | **Miscellaneous**  **(77,076)**  **2.69%** | | | **Leukemia**  **(66,036)**  **2.31%** | | | **Stomach**  **(59,846)**  **2.09%** | | |
|  |  | Rate | 95%CI | | Rate | 95%CI | | Rate | 95%CI | | Rate | 95%CI | | Rate | 95%CI | | Rate | 95%CI | | Rate | 95%CI | | Rate | 95%CI | | Rate | 95%CI | | Rate | 95%CI | |
|  |  |  | Upper | Lower |  | Upper | Lower |  | Upper | Lower |  | Upper | Lower |  | Upper | Lower |  | Upper | Lower |  | Upper | Lower |  | Upper | Lower |  | Upper | Lower |  | Upper | Lower |
| SEER 17 | 2000 | 719.1 | 725.8 | 712.4 | 252.4 | 248.5 | 256.4 | 252.5 | 256.6 | 248.5 | 155.6 | 158.8 | 152.4 | 81.7 | 84.0 | 79.4 | 75.5 | 77.7 | 73.3 | 51.1 | 52.9 | 49.3 | 30.1 | 31.6 | 28.8 | 51.2 | 52.0 | 48.3 | 45.8 | 47.6 | 44.1 |
|  | 2001 | 730.8 | 737.5 | 724.2 | 250.1 | 246.2 | 254.1 | 251.6 | 255.7 | 247.6 | 157.9 | 161.1 | 154.7 | 87.0 | 89.4 | 84.7 | 77.2 | 79.4 | 75.0 | 53.2 | 55.0 | 51.4 | 58.5 | 60.5 | 56.6 | 54.1 | 54.6 | 50.9 | 44.6 | 46.3 | 42.9 |
|  | 2002 | 733.0 | 739.7 | 726.4 | 242.0 | 238.2 | 245.8 | 241.2 | 245.1 | 237.3 | 153.7 | 156.8 | 150.5 | 87.7 | 90.1 | 85.4 | 79.5 | 81.8 | 77.3 | 55.1 | 56.9 | 53.3 | 55.3 | 57.2 | 53.4 | 52.0 | 52.5 | 49.0 | 44.4 | 46.1 | 42.7 |
|  | 2003 | 659.4 | 665.6 | 653.2 | 238.3 | 234.5 | 242.0 | 233.3 | 237.1 | 229.6 | 155.6 | 158.7 | 152.5 | 89.9 | 92.2 | 87.5 | 80.1 | 82.3 | 77.9 | 56.7 | 58.5 | 54.9 | 59.2 | 61.1 | 57.3 | 51.8 | 52.3 | 48.8 | 42.4 | 44.1 | 40.8 |
|  | 2004 | 655.8 | 661.9 | 649.7 | 234.5 | 230.9 | 238.3 | 223.4 | 227.1 | 219.7 | 161.4 | 164.6 | 158.3 | 96.3 | 98.7 | 93.9 | 81.7 | 83.9 | 79.5 | 57.3 | 59.1 | 55.5 | 60.1 | 62.0 | 58.2 | 51.4 | 52.0 | 48.5 | 43.4 | 45.0 | 41.7 |
|  | 2005 | 618.2 | 624.1 | 612.3 | 232.1 | 228.4 | 235.7 | 217.7 | 221.2 | 214.1 | 157.6 | 160.7 | 154.6 | 102.3 | 104.8 | 99.9 | 84.9 | 87.2 | 82.7 | 59.9 | 61.8 | 58.1 | 57.6 | 59.4 | 55.7 | 49.7 | 50.3 | 46.9 | 41.3 | 42.9 | 39.7 |
|  | 2006 | 666.9 | 673.0 | 660.9 | 233.3 | 229.7 | 237.0 | 208.7 | 212.2 | 205.2 | 157.5 | 160.6 | 154.5 | 102.4 | 104.8 | 100.0 | 82.2 | 84.4 | 80.0 | 61.3 | 63.1 | 59.4 | 60.8 | 62.8 | 59.0 | 49.1 | 49.8 | 46.5 | 40.0 | 41.5 | 38.5 |
|  | 2007 | 685.4 | 691.5 | 679.4 | 230.1 | 226.6 | 233.7 | 205.6 | 209.1 | 202.3 | 157.4 | 160.4 | 154.4 | 106.5 | 108.9 | 104.0 | 84.7 | 86.9 | 82.5 | 64.3 | 66.2 | 62.5 | 60.5 | 62.4 | 58.6 | 50.0 | 50.7 | 47.3 | 40.3 | 41.9 | 38.9 |
|  | 2008 | 623.4 | 629.1 | 617.8 | 225.3 | 221.9 | 228.9 | 200.7 | 204.1 | 197.4 | 154.1 | 157.1 | 151.2 | 111.1 | 113.5 | 108.6 | 83.8 | 85.9 | 81.7 | 65.7 | 67.6 | 63.9 | 59.6 | 61.5 | 57.8 | 51.5 | 52.1 | 48.8 | 38.4 | 39.9 | 37.0 |
|  | 2009 | 603.2 | 608.7 | 597.8 | 224.9 | 221.4 | 228.3 | 188.0 | 191.2 | 184.9 | 150.8 | 153.7 | 148.0 | 111.4 | 113.8 | 109.0 | 83.0 | 85.1 | 80.9 | 64.7 | 66.5 | 62.9 | 57.9 | 59.7 | 56.1 | 50.5 | 51.0 | 47.8 | 39.7 | 41.1 | 38.2 |
|  | 2010 | 573.6 | 578.9 | 568.4 | 219.2 | 215.8 | 222.6 | 177.1 | 180.2 | 174.1 | 153.4 | 156.3 | 150.5 | 112.8 | 115.3 | 110.4 | 79.2 | 81.3 | 77.2 | 61.9 | 63.7 | 60.2 | 58.3 | 60.1 | 56.6 | 48.2 | 48.6 | 45.5 | 38.7 | 40.1 | 37.3 |
|  | 2011 | 565.6 | 570.8 | 560.5 | 212.2 | 209.0 | 215.5 | 171.6 | 174.5 | 168.6 | 148.7 | 151.5 | 145.9 | 114.0 | 116.5 | 111.7 | 72.3 | 74.2 | 70.4 | 62.1 | 63.8 | 60.4 | 55.1 | 56.8 | 53.4 | 45.7 | 46.0 | 43.0 | 37.4 | 38.8 | 36.0 |
|  | 2012 | 466.4 | 471.0 | 461.8 | 208.8 | 205.6 | 212.0 | 164.0 | 166.9 | 161.2 | 149.6 | 152.4 | 146.8 | 117.6 | 120.0 | 115.2 | 71.8 | 73.6 | 69.9 | 64.4 | 66.1 | 62.7 | 52.0 | 53.6 | 50.4 | 43.3 | 43.5 | 40.6 | 37.5 | 38.8 | 36.1 |
|  | 2013 | 445.7 | 450.1 | 441.3 | 204.4 | 201.3 | 207.5 | 158.0 | 160.7 | 155.3 | 143.7 | 146.4 | 141.1 | 123.4 | 125.8 | 121.0 | 68.2 | 70.0 | 66.4 | 65.0 | 66.8 | 63.4 | 50.4 | 52.0 | 48.8 | 40.2 | 40.5 | 37.8 | 36.3 | 37.6 | 35.0 |
|  | 2014 | 406.9 | 411.0 | 402.8 | 199.0 | 196.0 | 202.0 | 158.0 | 160.7 | 155.4 | 142.7 | 145.3 | 140.1 | 129.0 | 131.4 | 126.6 | 65.1 | 66.9 | 63.4 | 64.4 | 66.1 | 62.8 | 46.2 | 47.7 | 44.8 | 35.1 | 35.5 | 33.0 | 37.1 | 38.5 | 35.8 |
|  | 2015 | 424.9 | 429.1 | 420.7 | 196.6 | 193.6 | 199.5 | 151.2 | 153.8 | 148.7 | 139.7 | 142.3 | 137.2 | 126.8 | 129.2 | 124.4 | 61.4 | 63.1 | 59.7 | 65.3 | 67.0 | 63.7 | 44.3 | 45.7 | 42.9 | 34.4 | 34.7 | 32.2 | 35.9 | 37.2 | 34.6 |
|  | 2016 | 445.7 | 450.0 | 441.6 | 191.3 | 188.5 | 194.2 | 146.6 | 149.1 | 144.1 | 136.2 | 138.7 | 133.8 | 125.7 | 128.1 | 123.4 | 54.5 | 56.1 | 53.0 | 65.8 | 67.5 | 64.2 | 42.1 | 43.5 | 40.7 | 31.7 | 31.7 | 29.4 | 33.4 | 34.7 | 32.2 |
|  | 2017 | 466.2 | 470.4 | 462.0 | 191.8 | 189.0 | 194.6 | 139.8 | 142.2 | 137.4 | 136.5 | 138.9 | 134.0 | 126.4 | 128.8 | 124.1 | 55.4 | 56.9 | 53.9 | 68.1 | 69.7 | 66.5 | 40.7 | 42.0 | 39.4 | 29.9 | 29.9 | 27.7 | 34.4 | 35.6 | 33.2 |
|  | 2018 | 462.5 | 466.7 | 458.4 | 181.0 | 178.4 | 183.8 | 138.6 | 141.0 | 136.3 | 130.8 | 133.2 | 128.5 | 123.5 | 125.8 | 121.3 | 52.2 | 53.7 | 50.8 | 66.2 | 67.8 | 64.6 | 39.2 | 40.5 | 37.9 | 28.4 | 28.6 | 26.5 | 33.0 | 34.1 | 31.8 |
|  | 2019 | 479.0 | 483.1 | 474.8 | 179.6 | 177.0 | 182.3 | 137.7 | 140.0 | 135.4 | 128.3 | 130.6 | 126.0 | 125.2 | 127.4 | 122.9 | 51.7 | 53.2 | 50.3 | 67.5 | 69.1 | 66.0 | 38.3 | 39.6 | 37.1 | 26.2 | 26.3 | 24.3 | 31.5 | 32.6 | 30.4 |
|  | 2000-2019 | 555.5 | 556.7 | 554.4 | 213.3 | 212.6 | 214.1 | 182.3 | 183.0 | 181.6 | 147.0 | 147.6 | 146.4 | 112.2 | 112.8 | 111.7 | 70.5 | 70.9 | 70.1 | 62.7 | 63.0 | 62.3 | 50.4 | 50.8 | 50.1 | 41.2 | 41.5 | 40.9 | 38.1 | 38.4 | 37.8 |
| APC  95%CI | - | -3.0*  -2.3, -3.6 | | | -1.7*  -1.6, -1.9 | | | -3.4*  -3.3, -3.6 | | | -1.1*  -0.8, -1.3 | | | 2.2*  2.6, 1.8 | | | -2.5*  -1.7, -3.2 | | | 1.1*  1.4, 0.8 | | | -1.9*  -0.7, -3.2 | | | -3.5*  -2.7, -4.2 | | | -1.7*  -1.6, -1.9 | | |

Rates are per 100,000 and age-adjusted to the 2000 US Std Population (19 age groups - Census P25-1130) standard.

APC, Annual percentage change. CI, Confidence intervals. *, p < 0.05.

**Supplementary Table 3.3. Top 10 cancers with the highest age-adjusted incidence rates in females aged ≥55 years during 2000–2019**

| **Registry** | **Year** | **Annual age-adjusted incidence rate** | | | | | | | | | | | | | | | | | | | | | | | | | | | | | |
| --- | --- | --- | --- | --- | --- | --- | --- | --- | --- | --- | --- | --- | --- | --- | --- | --- | --- | --- | --- | --- | --- | --- | --- | --- | --- | --- | --- | --- | --- | --- | --- |
|  |  | **Breast**  **(760,929)**  **34.42%** | | | **Lung and Bronchus**  **(297,903)**  **12.30%** | | | **Colon and Rectum**  **(271,173)**  **9.96%** | | | **Corpus Uteri**  **(168,863)**  **6.20%** | | | **Lymphoma**  **(99,150)**  **3.64%** | | | **Skin excluding Basal and Squamous**  **(98,416)**  **3.62%** | | | **Urinary Bladder**  **(69,616)**  **2.56%** | | | **Miscellaneous**  **(65,222)**  **2.40%** | | | **Ovary**  **(61,603)**  **2.26%** | | | **Kidney and Renal Pelvis**  **(60,742)**  **2.23%** | | |
|  |  | Rate | 95%CI | | Rate | 95%CI | | Rate | 95%CI | | Rate | 95%CI | | Rate | 95%CI | | Rate | 95%CI | | Rate | 95%CI | | Rate | 95%CI | | Rate | 95%CI | | Rate | 95%CI | |
|  |  |  | Upper | Lower |  | Upper | Lower |  | Upper | Lower |  | Upper | Lower |  | Upper | Lower |  | Upper | Lower |  | Upper | Lower |  | Upper | Lower |  | Upper | Lower |  | Upper | Lower |
| SEER 17 | 2000 | 394.3 | 5.1 | 5.1 | 147.0 | 149.7 | 144.4 | 179.1 | 182.0 | 176.2 | 76.3 | 79.8 | 76.0 | 55.7 | 57.3 | 54.1 | 35.9 | 37.2 | 34.6 | 37.4 | 38.8 | 36.1 | 22.8 | 23.9 | 21.8 | 37.2 | 38.6 | 35.9 | 23.7 | 38.6 | 35.9 |
|  | 2001 | 398.0 | 4.9 | 4.9 | 148.5 | 151.2 | 145.9 | 174.8 | 177.6 | 172.0 | 79.1 | 82.6 | 78.7 | 55.7 | 57.3 | 54.1 | 38.5 | 39.9 | 37.2 | 36.8 | 38.1 | 35.5 | 38.6 | 39.9 | 37.2 | 36.6 | 37.9 | 35.3 | 25.1 | 37.9 | 35.3 |
|  | 2002 | 389.3 | 4.3 | 4.3 | 148.5 | 151.1 | 145.9 | 171.8 | 174.6 | 169.1 | 75.4 | 78.5 | 74.7 | 55.6 | 57.2 | 54.0 | 38.8 | 40.1 | 37.5 | 36.7 | 38.1 | 35.5 | 36.5 | 37.8 | 35.2 | 36.2 | 37.6 | 35.0 | 25.7 | 37.6 | 35.0 |
|  | 2003 | 358.5 | 5.1 | 5.1 | 149.4 | 152.1 | 146.9 | 167.0 | 169.7 | 164.3 | 72.9 | 76.2 | 72.6 | 56.5 | 58.1 | 54.9 | 38.0 | 39.3 | 36.7 | 37.5 | 38.8 | 36.2 | 38.3 | 39.6 | 37.0 | 34.4 | 35.6 | 33.1 | 27.9 | 35.6 | 33.1 |
|  | 2004 | 357.2 | 4.7 | 4.7 | 151.0 | 153.6 | 148.4 | 159.2 | 161.9 | 156.6 | 72.5 | 75.6 | 72.0 | 58.8 | 60.4 | 57.2 | 40.8 | 42.2 | 39.5 | 39.4 | 40.7 | 38.1 | 38.9 | 40.2 | 37.6 | 33.2 | 34.4 | 32.0 | 27.8 | 34.4 | 32.0 |
|  | 2005 | 355.1 | 5.1 | 5.1 | 152.6 | 155.3 | 150.1 | 154.7 | 157.3 | 152.2 | 74.4 | 77.6 | 74.0 | 56.4 | 58.0 | 54.9 | 42.9 | 44.2 | 41.5 | 37.0 | 38.3 | 35.7 | 37.7 | 39.0 | 36.5 | 32.2 | 33.4 | 31.0 | 28.6 | 33.4 | 31.0 |
|  | 2006 | 355.7 | 4.8 | 4.8 | 152.3 | 154.9 | 149.7 | 151.9 | 154.5 | 149.4 | 74.8 | 77.9 | 74.3 | 57.9 | 59.5 | 56.4 | 42.1 | 43.5 | 40.8 | 35.3 | 36.5 | 34.1 | 38.4 | 39.7 | 37.1 | 33.5 | 34.7 | 32.3 | 30.5 | 34.7 | 32.3 |
|  | 2007 | 365.6 | 5.5 | 5.5 | 155.2 | 157.8 | 152.6 | 146.6 | 149.1 | 144.1 | 76.7 | 79.7 | 76.2 | 57.8 | 59.4 | 56.3 | 44.7 | 46.1 | 43.4 | 35.4 | 36.7 | 34.2 | 38.8 | 40.1 | 37.5 | 31.3 | 32.5 | 30.2 | 31.0 | 32.5 | 30.2 |
|  | 2008 | 370.0 | 5.2 | 5.2 | 155.2 | 157.7 | 152.6 | 144.3 | 146.8 | 141.9 | 76.9 | 80.2 | 76.6 | 58.3 | 59.8 | 56.7 | 46.0 | 47.4 | 44.6 | 36.1 | 37.3 | 34.8 | 38.4 | 39.7 | 37.2 | 32.6 | 33.7 | 31.4 | 31.3 | 33.7 | 31.4 |
|  | 2009 | 370.9 | 4.9 | 4.9 | 157.3 | 159.9 | 154.8 | 137.6 | 140.0 | 135.3 | 81.0 | 84.6 | 81.0 | 57.5 | 59.1 | 56.0 | 46.4 | 47.8 | 45.1 | 35.7 | 36.9 | 34.5 | 37.1 | 38.3 | 35.9 | 31.2 | 32.4 | 30.1 | 31.0 | 32.4 | 30.1 |
|  | 2010 | 362.0 | 5.2 | 5.2 | 150.1 | 152.6 | 147.7 | 129.7 | 132.0 | 127.5 | 81.5 | 84.9 | 81.4 | 54.3 | 55.8 | 52.8 | 48.1 | 49.4 | 46.7 | 35.0 | 36.2 | 33.8 | 35.2 | 36.4 | 34.1 | 30.5 | 31.7 | 29.5 | 29.9 | 31.7 | 29.5 |
|  | 2011 | 369.2 | 5.1 | 5.1 | 151.3 | 153.8 | 148.9 | 125.1 | 127.3 | 122.9 | 80.2 | 84.0 | 80.5 | 49.9 | 51.3 | 48.5 | 45.7 | 47.0 | 44.4 | 34.1 | 35.3 | 33.0 | 33.9 | 35.0 | 32.7 | 29.2 | 30.3 | 28.1 | 30.4 | 30.3 | 28.1 |
|  | 2012 | 368.4 | 5.0 | 5.0 | 151.4 | 153.9 | 149.0 | 120.6 | 122.7 | 118.5 | 84.1 | 87.9 | 84.4 | 48.1 | 49.4 | 46.7 | 48.5 | 49.9 | 47.2 | 34.9 | 36.1 | 33.8 | 32.7 | 33.9 | 31.6 | 28.7 | 29.8 | 27.7 | 31.1 | 29.8 | 27.7 |
|  | 2013 | 368.7 | 4.9 | 4.9 | 149.6 | 152.0 | 147.2 | 115.6 | 117.7 | 113.5 | 83.2 | 86.8 | 83.3 | 44.3 | 45.6 | 43.0 | 51.2 | 52.6 | 49.9 | 32.9 | 34.0 | 31.8 | 31.3 | 32.4 | 30.3 | 28.2 | 29.3 | 27.2 | 29.8 | 29.3 | 27.2 |
|  | 2014 | 368.4 | 5.0 | 5.0 | 148.5 | 150.9 | 146.2 | 114.3 | 116.4 | 112.3 | 85.0 | 88.7 | 85.3 | 43.6 | 44.8 | 42.3 | 53.7 | 55.1 | 52.4 | 32.8 | 33.9 | 31.8 | 28.1 | 29.1 | 27.1 | 28.3 | 29.3 | 27.3 | 30.1 | 29.3 | 27.3 |
|  | 2015 | 372.3 | 5.0 | 5.0 | 149.1 | 151.4 | 146.8 | 109.2 | 111.1 | 107.2 | 84.4 | 88.3 | 84.9 | 41.2 | 42.4 | 40.0 | 55.3 | 56.7 | 54.0 | 32.0 | 33.1 | 30.9 | 27.3 | 28.3 | 26.3 | 28.3 | 29.2 | 27.3 | 31.9 | 29.2 | 27.3 |
|  | 2016 | 369.0 | 4.9 | 4.9 | 146.8 | 149.0 | 144.5 | 107.9 | 109.8 | 106.0 | 88.0 | 91.9 | 88.5 | 37.9 | 39.0 | 36.7 | 53.3 | 54.7 | 52.0 | 32.5 | 33.6 | 31.5 | 27.4 | 28.3 | 26.4 | 26.2 | 27.2 | 25.3 | 31.3 | 27.2 | 25.3 |
|  | 2017 | 370.9 | 4.7 | 4.7 | 149.9 | 152.2 | 147.7 | 104.9 | 106.7 | 103.0 | 88.3 | 92.2 | 88.8 | 37.5 | 38.7 | 36.4 | 54.2 | 55.6 | 52.9 | 31.1 | 32.2 | 30.1 | 25.8 | 26.8 | 24.9 | 25.6 | 26.6 | 24.7 | 32.9 | 26.6 | 24.7 |
|  | 2018 | 373.5 | 5.2 | 5.2 | 143.8 | 146.0 | 141.7 | 103.1 | 105.0 | 101.3 | 87.7 | 91.5 | 88.2 | 35.6 | 36.7 | 34.6 | 54.9 | 56.3 | 53.6 | 31.3 | 32.3 | 30.3 | 25.0 | 25.9 | 24.1 | 24.3 | 25.2 | 23.5 | 31.5 | 25.2 | 23.5 |
|  | 2019 | 373.9 | 4.8 | 4.8 | 147.4 | 149.6 | 145.3 | 99.6 | 101.4 | 97.9 | 87.1 | 91.4 | 88.2 | 35.7 | 36.8 | 34.7 | 56.8 | 58.1 | 55.5 | 29.8 | 30.8 | 28.9 | 24.0 | 24.9 | 23.1 | 24.4 | 25.3 | 23.6 | 31.9 | 25.3 | 23.6 |
|  | 2000-2019 | 370.4 | 4.6 | 4.6 | 149.2 | 149.8 | 148.7 | 132.9 | 133.4 | 132.4 | 81.4 | 83.6 | 82.8 | 48.8 | 49.2 | 48.5 | 47.7 | 48.0 | 47.4 | 34.3 | 34.6 | 34.1 | 32.3 | 32.5 | 32.0 | 30.1 | 30.3 | 29.8 | 29.9 | 30.3 | 29.8 |
| APC  95%CI | - | -0.1  0.2, -0.3 | | | -0.1  0.0, -0.3 | | | -3.2*  -3.1, -3.3 | | | 1.0*  1.2, -0.8 | | | -2.7*  -2.7, -3.4 | | | 2.3*  2.6, 2.1 | | | -1.2*  -1.0, -1.4 | | | -2.2*  -1.1, -3.3 | | | -2.1*  -1.9, -2.3 | | | 1.1*  1.5, 0.7 | | |

Rates are per 100,000 and age-adjusted to the 2000 US Std Population (19 age groups - Census P25-1130) standard.

APC, Annual percentage change. CI, Confidence intervals. *, p < 0.05.

**Supplementary Table 4.1. The 1- to 5-year survival rates for patients with cancer aged 55 years and older (2000-2018)**

| Registry | Year | 1 year | | | 2 years | | | 3 years | | | 4 years | | | 5 years | | |
| --- | --- | --- | --- | --- | --- | --- | --- | --- | --- | --- | --- | --- | --- | --- | --- | --- |
|  |  | Rate | 95%CI | | Rate | 95%CI | | Rate | 95%CI | | Rate | 95%CI | | Rate | 95%CI | |
|  |  |  | Upper | Lower |  | Upper | Lower |  | Upper | Lower |  | Upper | Lower |  | Upper | Lower |
| SEER 17 | 2000 | 71.5 | 71.7 | 71.3 | 62.6 | 62.8 | 62.3 | 57.1 | 57.3 | 56.9 | 52.9 | 53.1 | 52.6 | 49.3 | 49.5 | 49.1 |
|  | 2001 | 72.0 | 72.2 | 71.8 | 63.3 | 63.5 | 63.1 | 57.8 | 58.0 | 57.6 | 53.7 | 54.0 | 53.5 | 50.2 | 50.4 | 50.0 |
|  | 2002 | 72.5 | 72.6 | 72.3 | 63.8 | 64.0 | 63.6 | 58.5 | 58.7 | 58.3 | 54.4 | 54.6 | 54.2 | 50.9 | 51.2 | 50.7 |
|  | 2003 | 72.1 | 72.3 | 71.9 | 63.4 | 63.6 | 63.2 | 58.0 | 58.2 | 57.8 | 53.9 | 54.1 | 53.6 | 50.4 | 50.6 | 50.1 |
|  | 2004 | 72.8 | 72.9 | 72.6 | 64.0 | 64.2 | 63.8 | 58.6 | 58.8 | 58.4 | 54.6 | 54.8 | 54.4 | 51.1 | 51.3 | 50.9 |
|  | 2005 | 73.0 | 73.1 | 72.8 | 64.4 | 64.6 | 64.2 | 59.1 | 59.3 | 58.9 | 55.1 | 55.3 | 54.8 | 51.6 | 51.8 | 51.4 |
|  | 2006 | 73.8 | 74.0 | 73.7 | 65.4 | 65.6 | 65.2 | 60.3 | 60.5 | 60.1 | 56.3 | 56.5 | 56.1 | 52.9 | 53.1 | 52.7 |
|  | 2007 | 74.5 | 74.7 | 74.3 | 66.3 | 66.5 | 66.1 | 61.3 | 61.5 | 61.1 | 57.3 | 57.5 | 57.1 | 54.0 | 54.2 | 53.8 |
|  | 2008 | 74.7 | 74.9 | 74.5 | 66.4 | 66.6 | 66.2 | 61.1 | 61.3 | 60.9 | 57.2 | 57.4 | 57.0 | 53.8 | 54.0 | 53.6 |
|  | 2009 | 75.2 | 75.4 | 75.0 | 66.8 | 67.0 | 66.6 | 61.7 | 61.9 | 61.5 | 57.8 | 58.0 | 57.6 | 54.5 | 54.7 | 54.3 |
|  | 2010 | 75.2 | 75.4 | 75.0 | 67.1 | 67.3 | 66.9 | 61.9 | 62.1 | 61.7 | 57.8 | 58.0 | 57.6 | 54.5 | 54.7 | 54.3 |
|  | 2011 | 75.9 | 76.1 | 75.7 | 67.7 | 67.9 | 67.5 | 62.5 | 62.7 | 62.3 | 58.5 | 58.7 | 58.3 | 55.2 | 55.4 | 55.0 |
|  | 2012 | 75.3 | 75.5 | 75.1 | 67.0 | 67.2 | 66.8 | 61.7 | 61.9 | 61.5 | 57.8 | 58.0 | 57.6 | 54.4 | 54.6 | 54.2 |
|  | 2013 | 75.4 | 75.6 | 75.3 | 67.1 | 67.3 | 66.9 | 61.9 | 62.1 | 61.7 | 57.9 | 58.1 | 57.7 | 54.5 | 54.7 | 54.3 |
|  | 2014 | 75.7 | 75.9 | 75.5 | 67.4 | 67.5 | 67.2 | 62.1 | 62.3 | 61.9 | 58.1 | 58.3 | 57.9 | 54.8 | 55.0 | 54.6 |
|  | 2015 | 76.4 | 76.6 | 76.2 | 68.3 | 68.4 | 68.1 | 63 | 62.8 | 61.4 | 59 | 59.2 | 58.8 | - | | |
|  | 2016 | 77 | 77.1 | 76.8 | 68.8 | 69.0 | 68.6 | 63.5 | 63.7 | 63.3 | - | | | - | | |
|  | 2017 | 77.4 | 77.6 | 77.3 | 69.3 | 69.5 | 69.1 | - | | | - | | | - | | |
|  | 2018 | 77.9 | 78.1 | 77.7 | - | | | - | | | - | | | - | | |
|  | 2000-2014 | 74.9 | 74.9 | 74.8 | 66.5 | 66.5 | 66.4 | 61.2 | 61.2 | 61.1 | 57.1 | 57.2 | 57.1 | 53.7 | 53.7 | 53.6 |

CI, Confidence intervals.

**Supplementary Table 4.2.1 The 5- years survival rates for the top 1-5 cancers with the highest incidence rates in patients older than 55 years (2000–2014)**

| **Registry** | **Year** | **Prostate** | | | **Breast** | | | **Skin excluding Basal and Squamous** | | | **Corpus Uterus** | | | **Urinary Bladder** | | |
| --- | --- | --- | --- | --- | --- | --- | --- | --- | --- | --- | --- | --- | --- | --- | --- | --- |
|  |  | Rate | 95%CI | | Rate | 95%CI | | Rate | 95%CI | | Rate | 95%CI | | Rate | 95%CI | |
|  |  |  | Upper | Lower |  | Upper | Lower |  | Upper | Lower |  | Upper | Lower |  | Upper | Lower |
| SEER 17 | 2000 | 79.5 | 79.9 | 79.1 | 75.6 | 76.1 | 75.1 | 70.4 | 71.5 | 69.2 | 68.6 | 69.8 | 67.3 | 59.0 | 60.0 | 58.0 |
|  | 2001 | 80.7 | 81.0 | 80.3 | 76.4 | 76.9 | 75.9 | 72.6 | 73.6 | 71.5 | 70.3 | 71.5 | 69.1 | 59.3 | 60.3 | 58.3 |
|  | 2002 | 81.4 | 81.7 | 81.0 | 77.1 | 77.6 | 76.6 | 72.3 | 73.3 | 71.2 | 68.7 | 69.9 | 67.4 | 59.0 | 59.9 | 58.0 |
|  | 2003 | 81.0 | 81.4 | 80.7 | 76.5 | 77.0 | 75.9 | 72.0 | 73.1 | 70.9 | 68.7 | 69.9 | 67.4 | 60.6 | 61.5 | 59.6 |
|  | 2004 | 82.0 | 82.4 | 81.6 | 76.5 | 77.0 | 76.0 | 72.0 | 73.0 | 71.0 | 69.6 | 68.3 | 66.8 | 59.9 | 60.9 | 59.0 |
|  | 2005 | 82.0 | 82.4 | 81.6 | 77.1 | 77.6 | 76.5 | 74 | 75.0 | 73.0 | 69.8 | 70.9 | 68.6 | 59.6 | 60.6 | 58.7 |
|  | 2006 | 83.5 | 83.9 | 83.2 | 77.9 | 78.3 | 77.4 | 73.2 | 74.2 | 72.2 | 70.8 | 72.0 | 69.7 | 60.6 | 61.5 | 59.6 |
|  | 2007 | 84.4 | 84.8 | 84.1 | 78.2 | 78.7 | 77.8 | 74.2 | 75.1 | 73.2 | 71.3 | 70.1 | 72.4 | 61.2 | 62.1 | 60.2 |
|  | 2008 | 84.2 | 84.5 | 83.8 | 78.6 | 79.1 | 78.1 | 74.4 | 75.3 | 73.5 | 71.4 | 72.5 | 70.3 | 60.3 | 61.2 | 59.3 |
|  | 2009 | 85.1 | 85.4 | 84.7 | 79 | 79.5 | 78.6 | 75.1 | 76.0 | 74.2 | 71.7 | 72.7 | 70.7 | 61.3 | 62.3 | 60.4 |
|  | 2010 | 85.2 | 85.6 | 84.9 | 78.9 | 79.3 | 78.4 | 75.3 | 76.1 | 74.4 | 72.5 | 73.5 | 71.5 | 60.6 | 61.5 | 59.7 |
|  | 2011 | 85.6 | 85.9 | 85.2 | 79.7 | 80.1 | 79.2 | 74.5 | 75.4 | 73.6 | 71.9 | 72.9 | 70.8 | 60.6 | 61.6 | 59.7 |
|  | 2012 | 84.5 | 84.9 | 84.2 | 79.7 | 80.1 | 79.2 | 76.7 | 77.5 | 75.8 | 72.4 | 73.4 | 71.4 | 60.8 | 61.7 | 59.9 |
|  | 2013 | 83.9 | 84.2 | 83.5 | 80.1 | 80.5 | 79.7 | 75.9 | 76.7 | 75.1 | 72.4 | 73.3 | 71.4 | 61.2 | 62.1 | 60.3 |
|  | 2014 | 83.3 | 83.7 | 82.9 | 80.4 | 80.9 | 80.0 | 76.9 | 77.6 | 76.1 | 72.6 | 73.5 | 71.6 | 61.3 | 62.2 | 60.4 |
|  | 2000-2014 | 83.1 | 83.2 | 83.0 | 78.7 | 78.8 | 78.5 | 75.0 | 75.2 | 74.8 | 71.4 | 71.6 | 71.1 | 60.6 | 62.4 | 60.4 |

CI, Confidence intervals.

**Supplementary Table 4.2.2 The 5- years survival rates for the top 6-10 cancers with the highest incidence rates in patients older than 55 years (2000–2014)**

| **Registry** | **Year** | **Kidney and Renal Pelvis** | | | **Lymphoma** | | | **Colon and Rectum** | | | **Pancreas** | | | **Lung and Bronchus** | | |
| --- | --- | --- | --- | --- | --- | --- | --- | --- | --- | --- | --- | --- | --- | --- | --- | --- |
|  |  | Rate | 95%CI | | Rate | 95%CI | | Rate | 95%CI | | Rate | 95%CI | | Rate | 95%CI | |
|  |  |  | Upper | Lower |  | Upper | Lower |  | Upper | Lower |  | Upper | Lower |  | Upper | Lower |
| SEER 17 | 2000 | 50.8 | 52.2 | 49.3 | 47.1 | 48.2 | 46.0 | 49.1 | 49.7 | 48.5 | 3.0 | 3.5 | 2.6 | 11.4 | 11.8 | 11.1 |
|  | 2001 | 51.5 | 52.9 | 50.1 | 49.6 | 50.7 | 48.5 | 49.4 | 50.0 | 48.8 | 3.3 | 3.8 | 2.9 | 11.6 | 12.0 | 11.3 |
|  | 2002 | 53.3 | 54.7 | 52.0 | 50.9 | 52.0 | 49.8 | 49.8 | 50.5 | 49.2 | 3.9 | 4.4 | 3.4 | 11.9 | 12.2 | 11.5 |
|  | 2003 | 53.3 | 54.6 | 52.0 | 52.9 | 54.0 | 51.9 | 50.5 | 51.2 | 49.9 | 3.0 | 3.5 | 2.6 | 12.1 | 12.5 | 11.8 |
|  | 2004 | 55.6 | 56.9 | 54.3 | 52.8 | 53.8 | 51.7 | 51.0 | 51.6 | 50.3 | 4.1 | 4.6 | 3.6 | 12.8 | 13.2 | 12.5 |
|  | 2005 | 57.8 | 59.0 | 56.5 | 54.3 | 55.3 | 53.3 | 51.9 | 52.6 | 51.3 | 4.2 | 4.7 | 3.7 | 13.4 | 13.7 | 13.0 |
|  | 2006 | 58.5 | 59.7 | 57.2 | 54.7 | 55.7 | 53.6 | 52 | 52.6 | 51.3 | 4.5 | 5.1 | 4.0 | 13.6 | 14.0 | 13.2 |
|  | 2007 | 59.8 | 61.0 | 58.6 | 55.5 | 56.5 | 54.5 | 53.3 | 53.9 | 52.6 | 4.7 | 5.2 | 4.2 | 14.1 | 14.5 | 13.7 |
|  | 2008 | 61.0 | 62.2 | 59.9 | 56.2 | 57.2 | 55.2 | 52.8 | 53.4 | 52.1 | 5.0 | 5.5 | 4.5 | 14.3 | 14.7 | 13.9 |
|  | 2009 | 61.3 | 62.4 | 60.1 | 56.6 | 57.6 | 55.7 | 53.3 | 54.0 | 52.6 | 5.5 | 6.1 | 5.0 | 14.8 | 15.2 | 14.4 |
|  | 2010 | 61.4 | 62.5 | 60.2 | 58.1 | 59.0 | 57.1 | 52.3 | 53.0 | 51.7 | 5.5 | 6.0 | 5.0 | 15.1 | 15.5 | 14.7 |
|  | 2011 | 61.0 | 62.1 | 59.9 | 57.5 | 58.5 | 56.5 | 52.2 | 52.9 | 51.5 | 6.6 | 7.2 | 6.0 | 15.9 | 16.4 | 15.5 |
|  | 2012 | 62.5 | 63.6 | 61.4 | 58.6 | 59.5 | 57.6 | 52.9 | 53.6 | 52.3 | 7.5 | 8.1 | 6.9 | 16.0 | 16.4 | 15.6 |
|  | 2013 | 62.6 | 63.7 | 61.5 | 59.0 | 60.0 | 58.0 | 52.5 | 53.1 | 51.8 | 7.7 | 8.3 | 7.1 | 16.8 | 17.2 | 16.4 |
|  | 2014 | 63.2 | 64.3 | 62.2 | 59.7 | 60.6 | 58.8 | 53.2 | 53.9 | 52.9 | 7.8 | 8.5 | 7.3 | 18 | 18.4 | 17.6 |
|  | 2000-2014 | 60.3 | 60.6 | 60.0 | 56.2 | 56.4 | 56.0 | 51.9 | 52.1 | 51.7 | 6.0 | 6.2 | 5.9 | 15.5 | 15.6 | 15.4 |

CI, Confidence intervals.

**Supplementary Table 5 Site Recode ICD-O-3/WHO 2008 Definition**

| Site Group | IICD-O-3 Site | ICD-O-3 Histology (Type) | Recode |
| --- | --- | --- | --- |
| ****Oral Cavity and Pharynx**** | | | |
| Lip | C000-C009 | excluding 9050-9055, 9140, 9590-9993 | 20010 |
| Tongue | C019-C029 |  | 20020 |
| Salivary Gland | C079-C089 |  | 20030 |
| Floor of Mouth | C040-C049 |  | 20040 |
| Gum and Other Mouth | C030-C039, C050-C059, C060-C069 |  | 20050 |
| Nasopharynx | C110-C119 |  | 20060 |
| Tonsil | C090-C099 |  | 20070 |
| Oropharynx | C100-C109 |  | 20080 |
| Hypopharynx | C129, C130-C139 |  | 20090 |
| Other Oral Cavity and Pharynx | C140, C142, C148 |  | 20100 |
| ****Digestive System**** | | | |
| Esophagus | C150-C159 | excluding 9050-9055, 9140, 9590-9993 | 21010 |
| Stomach | C160-C169 |  | 21020 |
| Small Intestine | C170-C179 |  | 21030 |
| Colon and Rectum | | | |
| Colon excluding Rectum | | | |
| Cecum | C180 | excluding 9050-9055, 9140, 9590-9993 | 21041 |
| Appendix | C181 |  | 21042 |
| Ascending Colon | C182 |  | 21043 |
| Hepatic Flexure | C183 |  | 21044 |
| Transverse Colon | C184 |  | 21045 |
| Splenic Flexure | C185 |  | 21046 |
| Descending Colon | C186 |  | 21047 |
| Sigmoid Colon | C187 |  | 21048 |
| Large Intestine, NOS | C188-C189, C260 |  | 21049 |
| Rectum and Rectosigmoidnction | | | |
| Rectosigmoid Junction | C199 | excluding 9050-9055, 9140, 9590-9993 | 21051 |
| Rectum | C209 |  | 21052 |
| Anus, Anal Canal and Anorectum | C210-C212, C218 |  | 21060 |
| Liver and Intrahepatic Bile Duct | | | |
| Liver | C220 | excluding 9050-9055, 9140, 9590-9993 | 21071 |
| Intrahepatic Bile Duct | C221 |  | 21072 |
| Gallbladder | C239 |  | 21080 |
| Other Biliary | C240-C249 |  | 21090 |
| Pancreas | C250-C259 |  | 21100 |
| Retroperitoneum | C480 |  | 21110 |
| Peritoneum, Omentum and Mesentery | C481-C482 |  | 21120 |
| Other Digestive Organs | C268-C269, C488 |  | 21130 |
| ****Respiratory System**** | | | |
| Nose, Nasal Cavity and Middle Ear | C300-C301, C310-C319 | excluding 9050-9055, 9140, 9590-9993 | 22010 |
| Larynx | C320-C329 |  | 22020 |
| Lung and Bronchus | C340-C349 |  | 22030 |
| Pleura | C384 |  | 22050 |
| Trachea, Mediastinum and Other Respiratory Organs | C339, C381-C383, C388, C390, C398, C399 |  | 22060 |
| ****Bones and Joints**** | C400-C419 | excluding 9050-9055, 9140, 9590-9993 | 23000 |
| ****Soft Tissue including Heart**** | C380, C470-C479, C490-C499 | excluding 9050-9055, 9140, 9590-9993 | 24000 |
| ****Skin excluding Basal and Squamous**** |  |  |  |
| Melanoma of the Skin | C440-C449 | 8720-8790 | 25010 |
| Other Non-Epithelial Skin | C440-C449 | excluding 8000-8005, 8010-8046, 8050-8084, 8090-8110, 8720-8790, 9050-9055, 9140, 9590-9993 | 25020 |
| ****Breast**** | C500-C509 | excluding 9050-9055, 9140, 9590-9993 | 26000 |
| ****Female Genital System**** | | | |
| Cervix Uteri | C530-C539 | excluding 9050-9055, 9140, 9590-9993 | 27010 |
| **Corpus and Uterus, NOS** | | | |
| Corpus Uteri | C540-C549 | excluding 9050-9055, 9140, 9590-9993 | 27020 |
| Uterus, NOS | C559 |  | 27030 |
| Ovary | C569 |  | 27040 |
| Vagina | C529 |  | 27050 |
| Vulva | C510-C519 |  | 27060 |
| Other Female Genital Organs | C570-C579, C589 |  | 27070 |
| ****Male Genital System**** | | | |
| Prostate | C619 | excluding 9050-9055, 9140, 9590-9993 | 28010 |
| Testis | C620-C629 |  | 28020 |
| Penis | C600-C609 |  | 28030 |
| Other Male Genital Organs | C630-C639 |  | 28040 |
| ****Urinary System**** | | | |
| Urinary Bladder | C670-C679 | excluding 9050-9055, 9140, 9590-9993 | 29010 |
| Kidney and Renal Pelvis | C649, C659 |  | 29020 |
| Ureter | C669 |  | 29030 |
| Other Urinary Organs | C680-C689 |  | 29040 |
| ****Eye and Orbit**** | C690-C699 | excluding 9050-9055, 9140, 9590-9993 | 30000 |
| ****Brain and Other Nervous System**** | | | |
| Brain | C710-C719 | excluding 9050-9055, 9140, 9530-9539, 9590-9993 | 31010 |
| Cranial Nerves Other Nervous System | C710-C719 | 9530-9539 | 31040 |
|  | C700-C709, C720-C729 | excluding 9050-9055, 9140, 9590-9993 |  |
| ****Endocrine System**** | | | |
| Thyroid | C739 | excluding 9050-9055, 9140, 9590-9993 | 32010 |
| Other Endocrine including Thymus | C379, C740-C749, C750-C759 |  | 32020 |
| ****Lymphoma**** | | | |
| **Hodgkin Lymphoma** | | | |
| Hodgkin - Nodal | C024, C098-C099, C111, C142, C379, C422, C770-C779 | 9650-9667 | 33011 |
| Hodgkin - Extranodal | All other sites |  | 33012 |
| **Non-Hodgkin Lymphoma** | | | |
| NHL - Nodal | C024, C098, C099, C111, C142, C379, C422, C770-C779 | 9590-9597, 9670-9671, 9673, 9675, 9678-9680, 9684, 9687-9691, 9695, 9698-9702, 9705, 9708-9709, 9712, 9714-9719, 9724-9729, 9735, 9737-9738, 9811-9818, 9823, 9827, 9837 | 33041 |
| NHL - Extranodal | All sites except C024, C098-C099, C111, C142, C379, C422, C770-C779 | 9590-9597, 9670-9671, 9673, 9675, 9678-9680, 9684, 9687, 9688, 9689-9691, 9695, 9698-9702, 9705, 9708-9709, 9712, 9714-9719, 9724-9729, 9735, 9737, 9738 | 33042 |
|  | All sites except C024, C098-C099, C111, C142, C379, C420-C422, C424, C770-C779 | 9811-9819, 9823, 9827, 9837 |  |
| ****Myeloma**** |  | 9731-9732, 9734 | 34000 |
| ****Leukemia**** | | | |
| Lymphocytic Leukemia | | | |
| Acute Lymphocytic Leukemia |  | 9826, 9835-9836 | 35011 |
|  | C420, C421, C424 | 9811-9819, 9837 |  |
| Chronic Lymphocytic Leukemia | C420, C421, C424 | 9823 | 35012 |
| Other Lymphocytic Leukemia |  | 9820, 9832-9834, 9940 | 35013 |
| Myeloid and Monocytic Leukemia | | | |
| Acute Myeloid Leukemia |  | 9840, 9861, 9865-9867, 9869, 9871-9874, 9877-9879, 9895-9897, 9898, 9910-9912, 9920 | 35021 |
| Acute Monocytic Leukemia |  | 9891 | 35031 |
| Chronic Myeloid Leukemia |  | 9863, 9875-9876, 9945-9946 | 35022 |
| Other Myeloid/Monocytic Leukemia |  | 9860, 9930 | 35023 |
| Other Leukemia | | | |
| Other Acute Leukemia |  | 9801, 9805-9809, 9931 | 35041 |
| Aleukemic, subleukemic and NOS |  | 9733, 9742, 9800, 9831, 9870, 9948, 9963-9964 | 35043 |
|  | C420, C421, C424 | 9827 |  |
| ****Mesothelioma**** |  | 9050-9055 | 36010 |
| ****Kaposi Sarcoma**** |  | 9140 | 36020 |
| ****Miscellaneous**** |  | 9740-9741, 9749-9769, 9950, 9960-9962, 9965-9968, 9970-9971, 9975, 9980, 9982-9987, 9989, 9991-9993 | 37000 |
|  | C760-C768, C809 | excluding 9050-9055, 9140, 9590-9993 |  |
|  | C420-C424 |  |  |
|  | C770-C779 |  |  |
| ****Invalid**** | Site or histology code not within valid range or site code not found in this table. | | 99999 |

This table is referenced from the SEER database. https://seer.cancer.gov/.
